# Cryo-EM and cryo-ET reveal the molecular architecture and host interactions of mycobacteriophage Douge

**DOI:** 10.1016/j.celrep.2025.116057

**Published:** 2025-07-24

**Authors:** Jitendra Maharana, Chun-Hsiung Wang, Li-An Tsai, Yi-Ting Liao, Cheng-Han Yang, Melvin C. Shen, Lourriel S. Macale, Thang Ngoc Tran, Joemark Narsico, Ronelito J. Perez, Sunil Kumar Tewary, Jian-Li Wu, Hong-You Lin, Shu-Wei Chang, Aaron Franklin, Patrick J. Moynihan, Deborah Jacobs-Sera, Krista G. Freeman, Graham F. Hatfull, Todd L. Lowary, Meng-Chiao Ho

**Affiliations:** 1 Institute of Biological Chemistry, Academia Sinica, Taipei, Taiwan; 2 Taiwan International Graduate Program in Chemical Biology and Molecular Biophysics (TIGP-CBMB), Academia Sinica, Taipei, Taiwan; 3 Institute of Bioinformatics and Structural Biology, College of Life Sciences and Medicine, National Tsing Hua University, Hsinchu, Taiwan; 4 Institute of Oral Medicine, National Cheng Kung University, Tainan, Taiwan; 5 Department of Civil Engineering, College of Engineering, National Taiwan University, Taipei, Taiwan; 6 Department of Biomedical Engineering, College of Engineering, National Taiwan University, Taipei, Taiwan; 7 School of Biosciences, University of Birmingham, Birmingham, UK; 8 Department of Biological Sciences, University of Pittsburgh, Pittsburgh, PA, USA; 9 Institute of Biochemical Sciences, National Taiwan University, Taipei, Taiwan; 10 Lead contact

## Abstract

Recent reports highlight the efficacy of engineered mycobacteriophages to treat non-tuberculosis mycobacterial disease. Molecular insights into mycobacteriophage architecture and host interactions could allow structure-guided phage engineering to increase efficacy and broaden host range, but such information is currently unavailable. We describe the cryoelectron microscopy (cryo-EM) structure of mycobacteriophage Douge, which contains 1,105 protein subunits assembled into a complete siphophage and is coated with glycan-binding domains for mycobacterial cell surface interactions. When filled with viral genome, the channel spanning the connector, tail, and baseplate is sealed by tape measure proteins, providing a genome gating system and requiring limited structural changes for genome ejection upon phage-host contact. Nanometer-resolution cryoelectron tomography (cryo-ET) snapshots of phage-host interactions show that the baseplate remains attached to the mycobacterial outer membrane during viral genome ejection. This study reveals high-resolution structural details of this mycobacteriophage and its interaction with host glycans.

## In brief

Maharana et al. employed cryo-EM, cryo-ET, and glycan array analyses to reveal the structural architecture and glycan-binding specificity of mycobacteriophage Douge as it infects its host, *Mycobacterium smegmatis*.

## Graphical Abstract

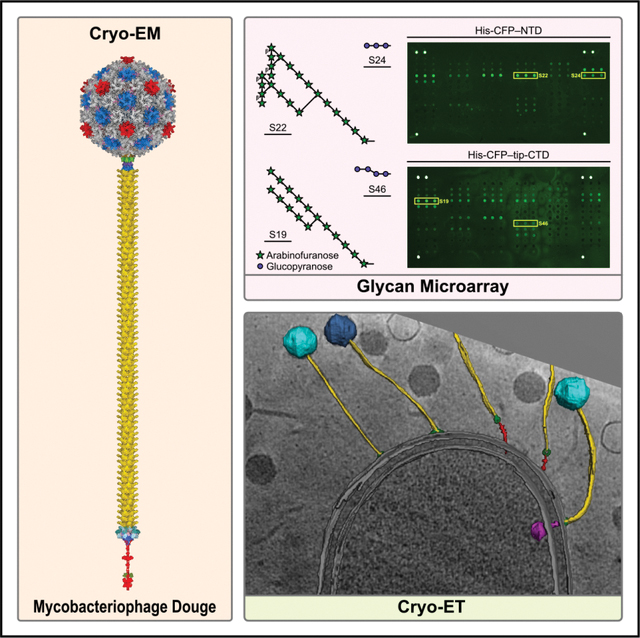

## INTRODUCTION

The *Mycobacterium* genus is heterogeneous, including species ranging from non-pathogenic to clinically significant obligate or opportunistic human pathogens.^1^ *Mycobacterium tuberculosis* causes tuberculosis, leading to over one million deaths annually, and the emergence of drug-resistant *M. tuberculosis* strains is a matter of continued concern.^2,3^ Non-tuberculous mycobacteria (NTMs), often intrinsically resistant to antimicrobial agents, are increasingly found in immunocompromised patients.^4–6^ Challenges in treating drug-resistant mycobacteria have motivated interest in alternative therapies.^6^ Recent studies have demonstrated the successful use of mycobacteriophage cocktails against NTM infections,^7–12^ highlighting the potential of phage therapy in treating mycobacterial diseases. Despite promising clinical studies, limited knowledge of mycobacteriophage molecular architecture and their host-binding machinery hinders structure-based engineering to improve potency or broaden host range.^13^ Over 14,400 mycobacteriophages have been isolated (http://phagesdb.org), and although numerous high-resolution cryoelectron microscopy (cryo-EM) phage capsid structures have been reported,^14^ only two complete mycobacteriophage structures are available: one at low (~30 Å) using EM^15^ and another at high resolution using cryo-EM.^16^

Most mycobacteriophages are siphophages, classified within class *Caudoviricetes.* Bacteriophages of this class possess a tail with a tip with host recognition capabilities, facilitating attachment to the host cell wall to deliver the viral genome.^17,18^ These double-stranded DNA (dsDNA) tailed phages are further classified based on tail morphology: myophages have a contractile tail; siphophages have a long, flexible, non-contractile tail; and podophages have a short, stubby tail.^19–24^ Previous structural studies have shown that myophages drill through the bacterial cell wall in a syringe-like fashion by contracting an outer protein sheath surrounding the tail tube, triggering conformational changes in connector proteins for viral genome release.^25–28^ Although siphophages represent over 60% of all tailed phages and most mycobacteriophages belong to this group,^18,21^ the molecular mechanisms underlying siphophage genome delivery lag behind what is known for myophages.

**Figure 1.**
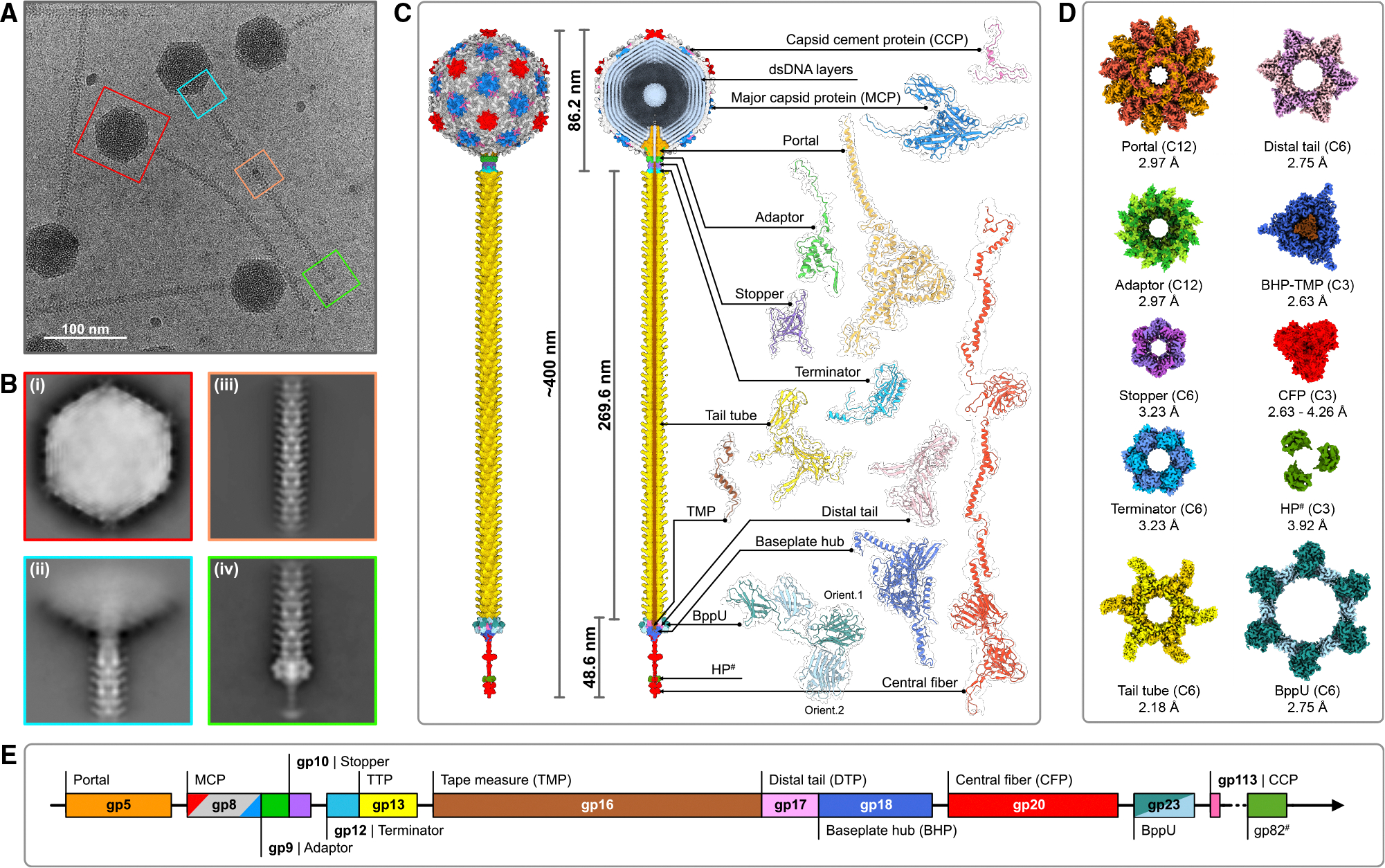
Cryo-EM structure of mycobacteriophage Douge (A) A representative micrograph showing the Douge morphology. Scale bar: 100 nm. (B) 2D class averages of Douge sub-components—(i) capsid, (ii) connector, (iii) tail tube, and (iv) baseplate—are highlighted in red, cyan, orange, and green boxes, respectively. (C) Composite cryo-EM map displays the complete phage, with color-coding based on the gene map in (E). Internal structural details, such as the dsDNA (pale blue), portal protein (orange), and tape measure protein (TMP) (brown), are shown in a z-clipped map, and the atomic coordinates of each proteinaceous component are displayed along with transparent segmented maps. (D) Top views of various phage protein symmetric rings, including portal, adaptor, stopper, terminator, tail tube (TTP), distal tail (DTP), baseplate hub (BHP), TMP, central fiber (CFP), and baseplate upper protein (BppU) and a hypothetic protein (HP^#^), likely gp82, are shown with corresponding ring symmetries and map resolutions. (E) Schematic representation of gene products (gp) encoding 13 structural proteins are color coded.

The modular architecture of siphophages has four sub-components: capsid, capsid-tail connector (connector), tail tube, and baseplate (Figures 1A–1C).^17^ The predominately icosahedral capsid is densely packed with viral dsDNA, generating internal pressures of tens of atmospheres.^29–32^ A unique 5-fold capsid vertex to which the tail is affixed occupies a dodecameric portal protein, serving as the entry/exit point for viral dsDNA to be packed during phage assembly and ejected upon infection.^20^ The connector, composed of portal, adaptor, stopper, and terminator proteins, forms a channel between the capsid and tail tube.^33–37^ Siphophages and phage-like particles display a wide range of tail lengths (20–875 nm) composed of 5–200 stacked trimeric or hexameric rings of tail tube proteins (TTPs).^23,36–41^ The baseplate, attached to the tail tube via distal tail proteins (DTPs), mediates host recognition and adsorption, thereby determining host specificity.

Despite growing interest in mycobacteriophages for disease treatment, significant knowledge gaps persist regarding siphophage structure and the molecular mechanisms of host recognition and genome delivery. Although siphophages share common structural features, their species specificity and host-binding mechanism likely arise from differences in three-dimensional (3D) architecture and protein composition. The underlying features that dictate how these differences impact mycobacteriophage-host interactions are poorly understood. These gaps arise from limited atomic-level siphophage structures and high-resolution data on phage-host interactions.

In this study, we isolated the mycobacteriophage Douge and determined its complete cryo-EM structure at 2.18–4.26 Å resolution, enabling full atomic model building. This allowed us to elucidate the intricate protein interaction networks that stabilize the molecular architecture of this siphophage. Structural comparisons of Douge with and without its genome suggest that limited structural changes accompany genome ejection and suggest a genome gating mechanism regulated by the release of tape measure proteins (TMPs). The Douge surface is densely coated with 952 glycan-binding domains that specifically recognize fragments of mycobacterial lipoarabinomannan/arabinomannan (LAM/AM), a major surface antigen,^42^ and effectively label the surface of *Mycobacterium smegmatis*. Cryoelectron tomography (cryo-ET) provided nanometer-resolution snapshots of how Douge interacts with the host before and after genome ejection. Taken together, the results of our study unveil the first atomic-resolution architecture of a mycobacteriophage, providing insights into the mechanisms underlying phage binding to the bacterial surface and genome release.

## RESULTS

### Overall structure of mycobacteriophage Douge

Genome sequencing revealed that Douge is related to 75 other siphophages within cluster L, specifically grouping in subcluster L4.^43^ Cryoelectron micrographs confirm the presence of the capsid, connector, tail tube, and baseplate (Figure 1A). However, due to the substantial size and flexibility of the tail tube, the reconstruction of the entire virion by cryo-EM was not feasible. To overcome this, we independently reconstructed the 3D structure of individual phage sub-components and assembled them into a composite map representing the complete virion (Figures 1A–1D, S1, and S2; Table S1).

The cryo-EM map of the capsid was reconstructed at 3.20 Å using icosahedral symmetry. Focused refinement enhanced the resolution of individual capsomeres (penton and hexons) to 2.80–2.95 Å (Figures S1A and S1B). The connector-vertex was reconstructed with C1 symmetry at 3.74 Å, followed by local reconstruction of the connector at 3.18 Å using C6 symmetry. Subsequent local refinements improved the resolution of the dodecameric portal-adaptor (C12 symmetry) and the hexameric stopper-terminator subcomplex (C6 symmetry) to 2.82 and 2.96 Å, respectively. The capsid vertex (C5 symmetry) was reconstructed at 3.24 Å (Figures S1C and S1D) and the tail tube (C6 symmetry) at 2.18 Å (Figures S2B and S2C). By employing C3 symmetry, the baseplate was reconstructed at 2.97 Å, followed by focused refinements in three segments: (1) tail tube with C6 symmetry, (2) baseplate ring composed of DTPs and baseplate upper proteins (BppUs) with C3 symmetry, and (3) distal baseplate, including TMPs, baseplate hub proteins (BHPs), and central fiber proteins (CFPs) with C3 symmetry. These refinements achieved resolutions of 2.71, 2.75, and 2.63 Å, respectively (Figures S2F and S2G). Finally, the distal flexible CFP, requiring a separate reconstruction, was resolved at 4.26 Å by employing C3 symmetry. Further focused refinement of its terminus yielded a resolution of 3.92 Å (Figures S2H and S2I), sufficient to build the structure of the CFP with the facilitation of the predicted model from AlphaFold2.^44^

Douge has a length of ~400 nm, featuring a capsid with diameters ranging from 71.3 to 77.9 nm, a connector, a 269.6-nm-long tail tube, and a 48.6 nm baseplate subcomplex (Figure 1C). Phage structure regions that were not fully resolved include (1) significant portions of the TMP, as only a short part of the C terminus could be traced in the baseplate region, (2) the genomic dsDNA, and (3) a hypothetical protein, attached to the CFP. The complete structure includes 13 distinct protein subunits, totaling 1,105 protein protomers (Figures 1C–1E).

### Capsid

**Figure 2.**
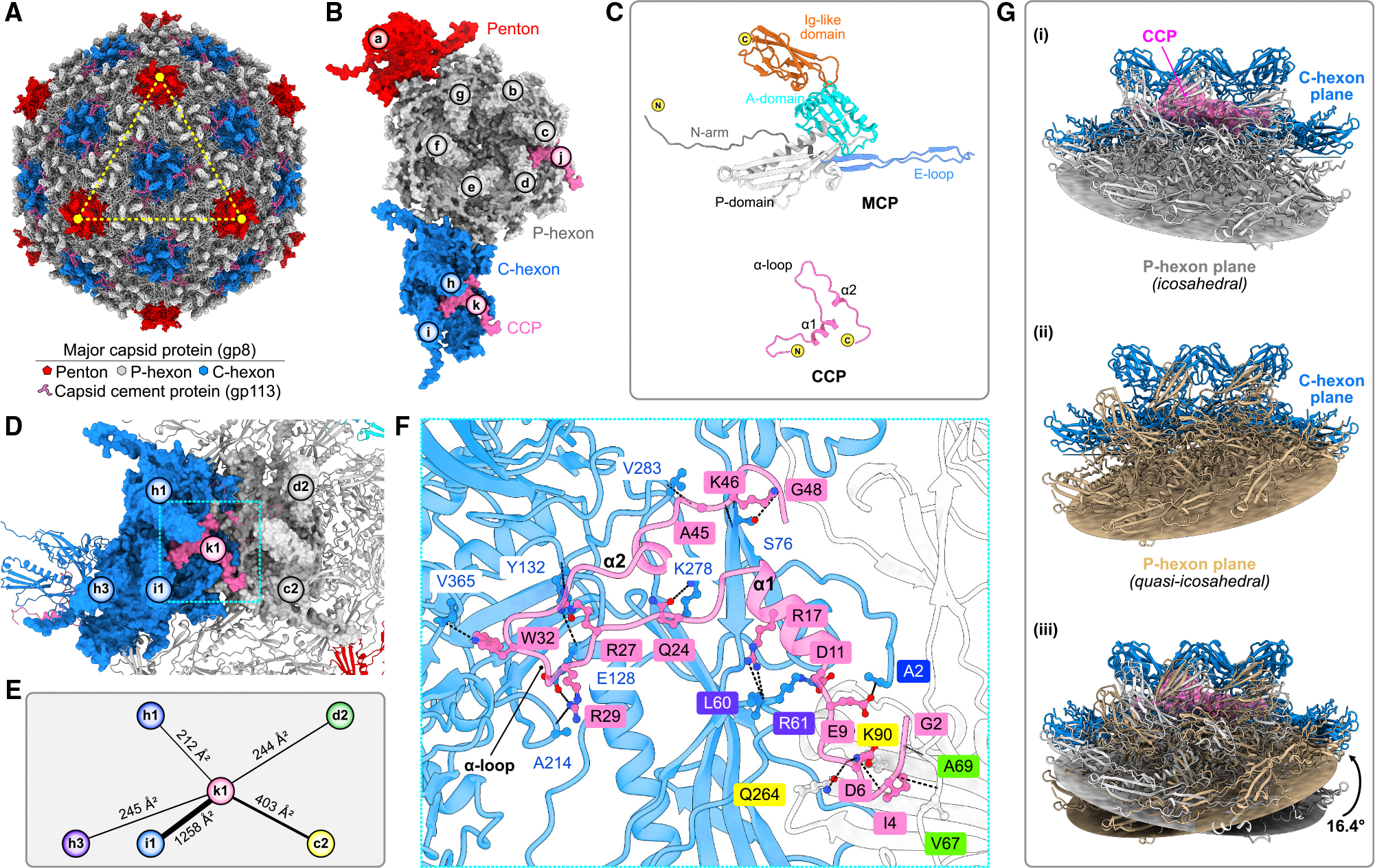
Overall capsid structure of mycobacteriophage Douge (A) Cryo-EM map of Douge capsid displayed along the icosahedral 3-fold axis, revealing the arrangement of major capsid proteins (MCPs) within three capsomeres, penton, P-hexon, and C-hexon, along with two capsid cement proteins (CCPs) at the 3-fold icosahedral plane. Penton, P-hexon, C-hexon, and CCPs are colored in red, blue, gray, and pink, respectively. (B) The icosahedral asymmetric unit of Douge capsid features a monomeric subunit of a penton, a complete P-hexon, and two monomeric C-hexon subunits with associated CCPs on the 3-fold axis. The nine MCPs and two CCPs in the asymmetric unit are labeled with letters (a)–(i) and (j) and (k), respectively. (C) Atomic coordinates of MCPs and CCPs are presented in colored ribbon models, with MCP domain regions highlighted in different colors. (D) Surface diagram showing the interaction of one CCP subunit with five MCP subunits. MCP/CCP subunits are labeled with letters (a)–(k) as in (B), and numbers denote the asymmetric units. (E) Buried surface areas between CCP and MCP subunits are depicted. (F) Detailed interactions between the CCP and neighboring MCP subunits, with interacting residues shown in ball-and-stick model and H-bonds as dotted black lines. (G) Comparison of the CCP-bound icosahedral C-hexon-P-hexon di-capsomere (i) and the CCP-free quasi-icosahedral C-hexon-P-hexon di-capsomere (ii) and their superimposed view based on the C-hexon (iii). Ribbon diagrams with corresponding planes reveal distinct spatial arrangements of C-hexon-P-hexon di-capsomeres with an angular difference of 16.4°.

Exhibiting T = 9 organization, the icosahedral capsid is composed of 535 major capsid proteins (MCPs) and 120 capsid cement proteins (CCPs). The assembly has 12 pentons at icosahedral vertices and 80 hexons across icosahedral faces, of which 20 central hexons (C-hexons) occupy the centers of icosahedral faces and 60 peripentonal hexons (P-hexons) are positioned along the icosahedral edges (Figures 2A–2C). One 5-fold vertex is replaced by a dodecameric ring of portal proteins. The icosahedral shell formed by MCP protomers encapsulates the phage genome.

The Douge MCP possesses a common HK97 fold,^14,45^ consisting of a long N-terminal arm (N-arm), a periphery domain (P-domain), an extended loop (E-loop), and an axial domain (A-domain), capped with a protruding immunoglobulin (Ig)-like domain at the C-terminal end, potentially enabling the incorporation of surface domains to the HK97 fold (Figure 2C; Table S2). As observed in other phages, capsid assembly is stabilized by both intra- and inter-capsomere interactions (Figures S3A–S3D).^46^ In Douge, intra-capsomere interactions are facilitated primarily by electrostatic complementarity with negatively charged interfaces of the A-domain of one subunit interacting with positively charged interfaces of neighboring A-domains and Ig-like domains (Figure S3E). Inter-capsomere interactions involve a combination of electrostatic and hydrophobic contacts (Figure S3F).

CCPs, composed of two short α helices and one α-loop, are present around the C-hexons on the icosahedral plane (Figures 2A–2C). Each CCP interacts with five MCP protomers, covering a total buried surface area of ~2,360 Å^2^ (Figures 2D and 2E). One CCP subunit forms prominent main chain-side chain H-bonds with a specific MCP subunit (Figure 2F). CCPs are restricted to the icosahedral plane and absent at the quasi-icosahedral plane (Figure 2A). Superimposition of an icosahedral C-hexon-P-hexon di-capsomere onto a quasi-icosahedral counterpart, aligned via C-hexons, reveals a 16.4° shift in the P-hexon orientation, explaining the exclusion CCP from the quasi-icosahedral plane (Figure 2G).

### Connector

**Figure 3.**
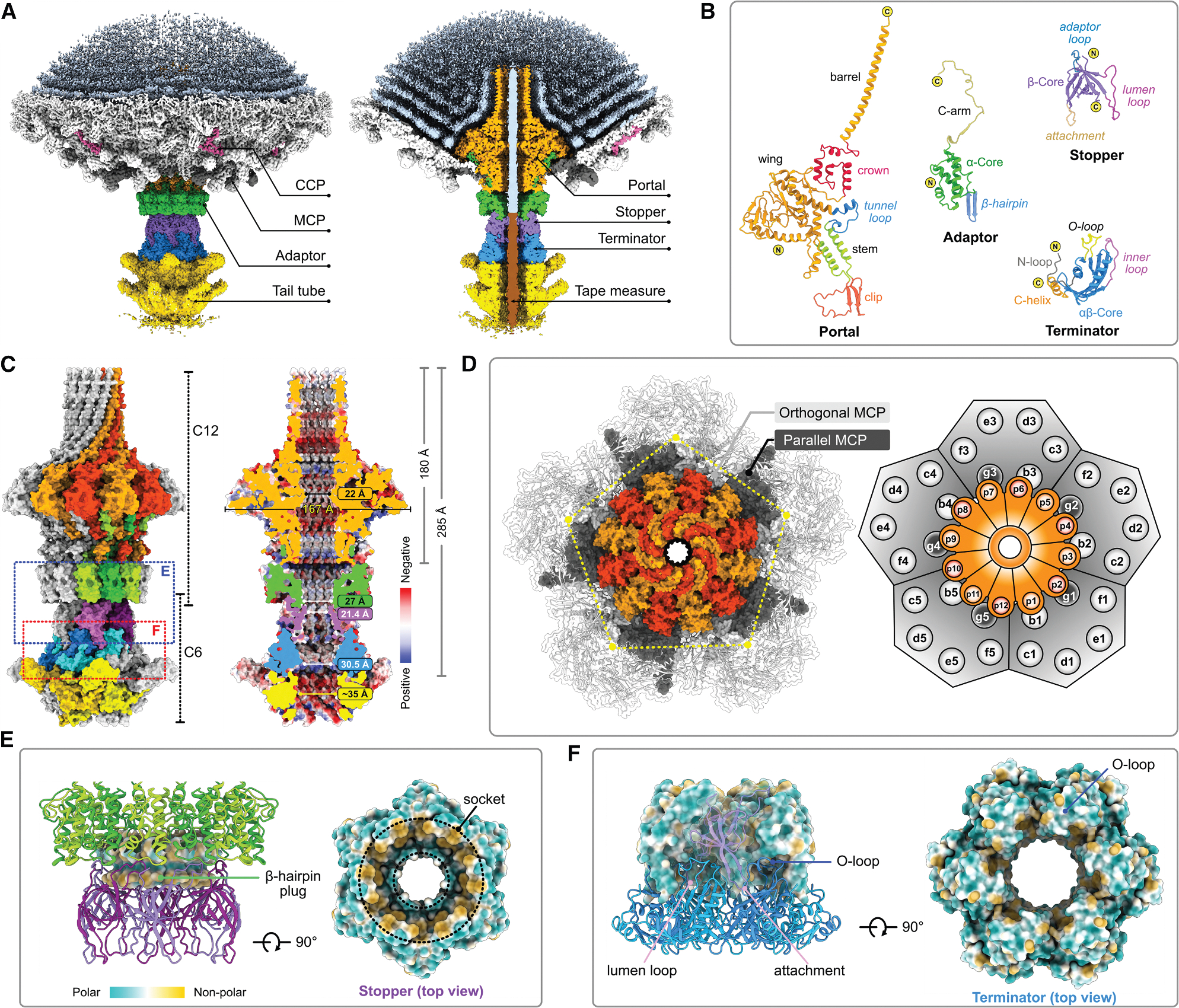
Connector structure (A) Composite cryo-EM map displaying the Douge connector and capsid vertex, with a cutaway map revealing internal components like the portal (orange), TMP (brown), and dsDNA (pale blue) layers. (B) Atomic structures of portal, adaptor, stopper, and terminator proteins shown as colored ribbon models, with structural domain regions highlighted. (C) Surface diagram illustrating arrangement of connector proteins in representative symmetries (left), with a z-clipped view displaying electrostatic surface potential across the connector channel, highlighting the inner diameter of each segment (right). (D) Top view and schematic of the portal-vertex showing symmetry-mismatched association between the 12-fold portal (orange) and 5-fold P-hexon (gray) vertex, with parallel and orthogonal MCP subunits highlighted. The 12 portals are labeled as p1–p12. The 6 MCPs in P-hexons are labeled as (b)–(g), and numbers 1–5 denote five distinct P-hexons. (E) Symmetry-mismatched assembly between dodecameric adaptor (green) and hexameric stopper (violet), with β-hairpin plug of the adaptor (left) and hydrophobic socket of the stopper (right) displayed using lipophilicity surfaces. (F) Hexameric assembly of stopper and terminator rings showing the interlocking loop system (left) and top view of the terminator ring in lipophilicity surface representation (right).

The connector, composed of portal-adaptor and stopper-terminator subcomplexes, has two functions: gatekeeping the viral genome and connecting the capsid and tail tube.^33,37,38,47^ Individual refinement of each subcomplex with symmetry followed by composite map generation revealed the dodecameric portal-adaptor subcomplex and a hexameric stopper-terminator subcomplex (Figures 3A–3C). The dodecameric portal, measuring 180 Å in height and 167 Å in outer diameter, is inserted into one 5-fold vertex of the icosahedral capsid (Figures 3C and 3D), consistent with observations in other bacteriophages.^37,48–51^ The composite map, combining a capsid-vertex map (3.24 Å) and a portal-adaptor map (2.82 Å), unravels detailed portal-capsid interactions.

Interaction analyses revealed that the α1 helix and β7-β8 loop (residues 187–196) of portal proteins and the C-arm (125–138) of adaptor proteins closely approach the channel of the 5-fold capsid vertex, creating a C5-C12 symmetry mismatch juncture between the vertex and portal-adaptor subcomplex (Figures 3D and S4A–S4C). Due to this C5-C12 symmetry mismatch, 12 portal and adaptor proteins interact with different MCP regions (Figure S4C). The C1 connector map revealed variable traces of the flexible C-arm (125–154) of the adaptor, suggesting that the C terminus adopts multiple conformations to intercalate in different capsid environments (Figure S4C). Overlaying the Cα trace from the C1 map further showed that the α1 helix and β7-β8 loop of the portal and the C-arm (125–154) of the adaptor adopt diverse conformations to compensate for the symmetry mismatch (Figures S4D and S4E). Such structural morphing to address symmetry mismatches has also been observed in other bacteriophages.^48,49,51^

Below the portal, adaptor proteins assemble into another dodecameric ring, with each C-arm (106–138) interacting with four portal protein subunits. The proximal C-arm region (106–116) is sandwiched between clip domains of two adjacent portal protomers (p1/p2), while the distal part (117–138) is positioned between the wing domains of two others (p3/p4). The adaptor-portal interactions are primarily electrostatic and involve eight side chain-side chain and four main chain-main chain H-bonds, along with several hydrophobic contacts, covering a total surface area of ~32,364 Å^2^ (Figures S5A–S5D; Table S3).

The stopper protein adopts an antiparallel β sheet fold and assembles into a hexameric ring (Figures 3B and 3E). The dodecameric β-hairpin plug (76–85) of the adaptor is firmly inserted into the socket formed by adaptor loops (89–101) of hexameric stoppers (Figure 3E), creating a C12-C6 symmetry mismatch juncture. The adaptor-stopper interaction is primarily hydrophobic, supplemented by π-π stacking and one H-bond (R77^a1^–D96^s1^) with an overall surface area of ~5,718 Å^2^ (Figures S5E and S5F). The terminator, positioned between the stopper and tail tube, forms a homo-hexamer ring. Interaction analyses unveiled an interlocking loop system connecting the stopper and terminator (Figure 3F), which includes the attachment (12–20) and lumen (33–54) loops of the stopper and the O-loop (35–50) and the inner loop (120–134) of the terminator. Specifically, the lumen loop and the exterior attachment loop of the stopper secure the terminator in place via H-bonds (Table S3) with additional cation-π and hydrophobic contacts, covering a total surface area of ~8,718 Å^2^ (Figures S5G and S5H). Thus, the connector forms a 285 Å channel with a slightly negatively charged inner surface, allowing the smooth passage of negatively charged dsDNA. The narrowed diameters of the portal, adaptor, stopper, and terminator lumens are 22, 27, 21.4, and 30.5 Å, respectively, wide enough for extended dsDNA to transit (Figure 3C).^52^

### Tail tube

**Figure 4.**
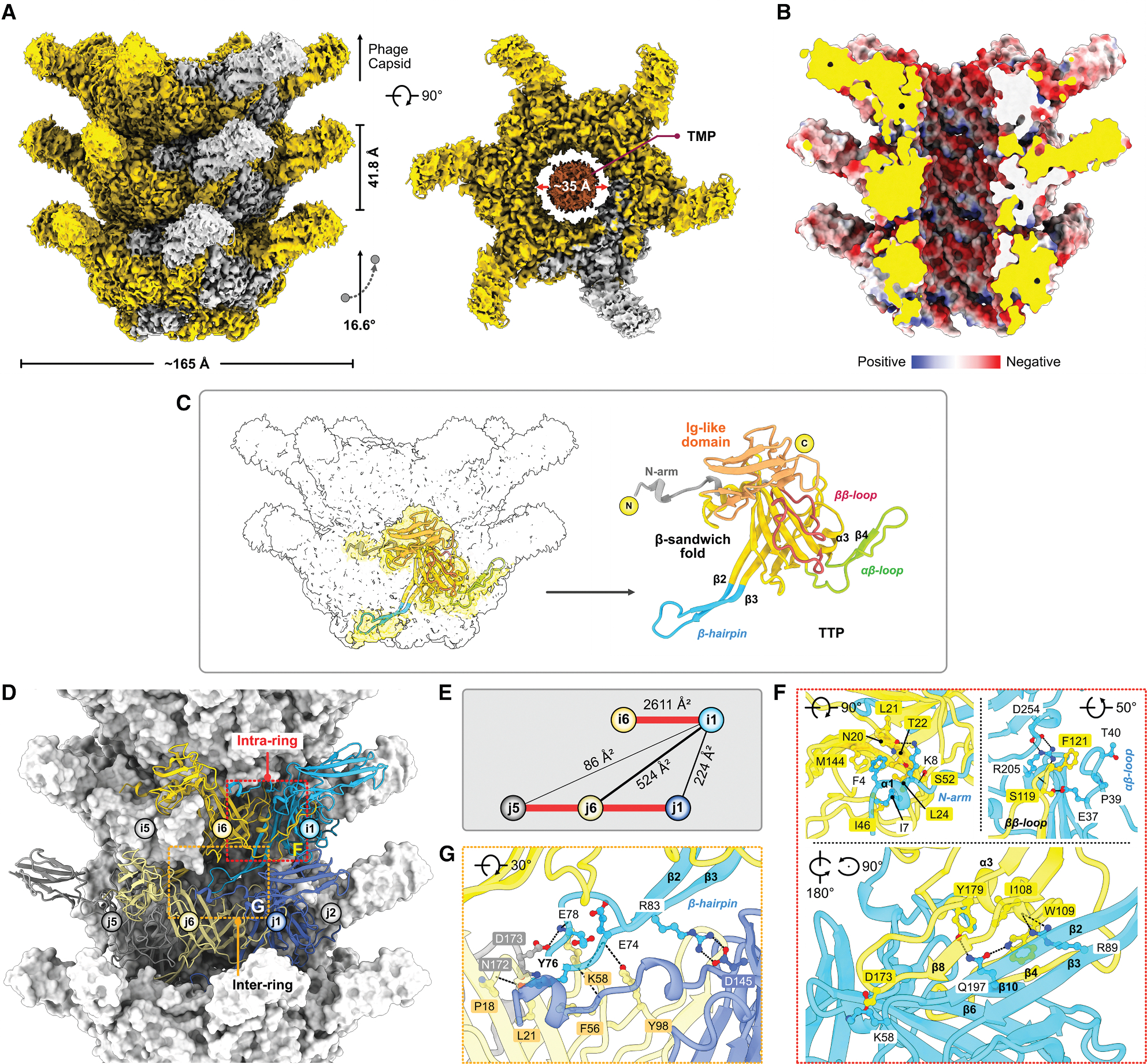
Structure of the tail tube (A) Cryo-EM map of the Douge tail tube at 2.18 Å resolution, displaying the helical arrangement of the TTPs, with one helical strand in gray, along with helical rise and twist information. The right image shows a top view of a single hexameric TTP ring, with the TMP density in brown within the tail tube channel. (B) Electrostatic surface distributions of the three-ring TTP structure (z-clipped view), revealing a negatively charged inner channel. (C) A single TTP subunit within the two-tier tail tube density, shown alongside its ribbon diagram highlighting key structural domain regions. (D) Overview of intra- and inter-ring interactions depicted with interface subunits in different cartoons. (E) Buried surface areas involved in each interface are shown with colored bars and values. (F and G) Detailed illustrations of (F) intra- and (G) inter-ring interactions between TTP subunits, with interacting residues shown as ball-and-stick models and H-bonds in dotted black lines. The interaction views were selected for optimal visibility. The approximate rotational angles along the axes, relative to (D), are now indicated in (F) and (G) for clarity.

Despite challenges posed by tail tube flexibility in achieving atomic resolution,^40^ we reconstructed the cryo-EM density map at 2.18 Å, clearly resolving side-chain density. The TTP rings form a six-start helix, with each ring rising 41.8 Å with a twist of 16.6° (Figure 4A). The tail tube elongates through the assembly of 64 layers around trimeric TMPs (Figure S2A), creating a six-stranded helical tube that is 269.6 nm long (Figure 1B). This structure possesses an outer diameter of ~165 Å and an inner channel diameter of ~35 Å (Figure 4A) that is lined with a negatively charged electrostatic surface that facilitates the passage of dsDNA.^22^ (Figure 4B). The proximal TTP hexamer is capped by a hexameric terminator ring, with each terminator protomer interacting with two TTP subunits. The interaction is predominately electrostatic, including multiple H-bonds and several hydrophobic contacts, encompassing a buried surface area of ~6,822 Å^2^ (Figures S5F–S5I; Table S3).

The Douge tail tube represents a minimal TTP system composed of an N-arm, a β-sandwich fold flanked by a central helix (α3), and αβ-, ββ-, and β-hairpin loops, forming a homo-hexameric ring with an additional C-terminal Ig-like domain protruding outward (Figure 4C; the structural similarities are listed in Table S2). Investigation of the tail tube assembly revealed two distinct interactions: an intra-ring mediated by the N-arm, ββ-loop, and αβ-loop and an inter-ring primarily involving the β-hairpin (Figure 4D). Intra-ring interactions are dominated by backbone H-bonds (between β2-β8 and β10-β4 of the i1 and i6 subunits) with additional electrostatic and hydrophobic contact networks, with a surface area of ~2,611 Å^2^ (Figures 4E and 4F). At the inter-ring interface, each TTP subunit interacts with three TTP subunits from the adjacent ring. The cumulative interaction surface area contributed by all six subunits of one ring interacting with the adjacent ring is ~5,004 Å^2^ (Figures 4D and 4E). This inter-ring interaction, crucial for the structural integrity of TTP ring layers, involves hydrophobic and electrostatic contributions, including a salt bridge (R83^i1^–D145^j1^), six main chain-side chain H-bonds (E74^i1^–K58/Y98^j6^, Y76^i1^–P18/F56/K58^j6^, and E78^i1^–K58^j6^), and several hydrophobic contacts (Figure 4G; Table S3).

### Baseplate

**Figure 5.**
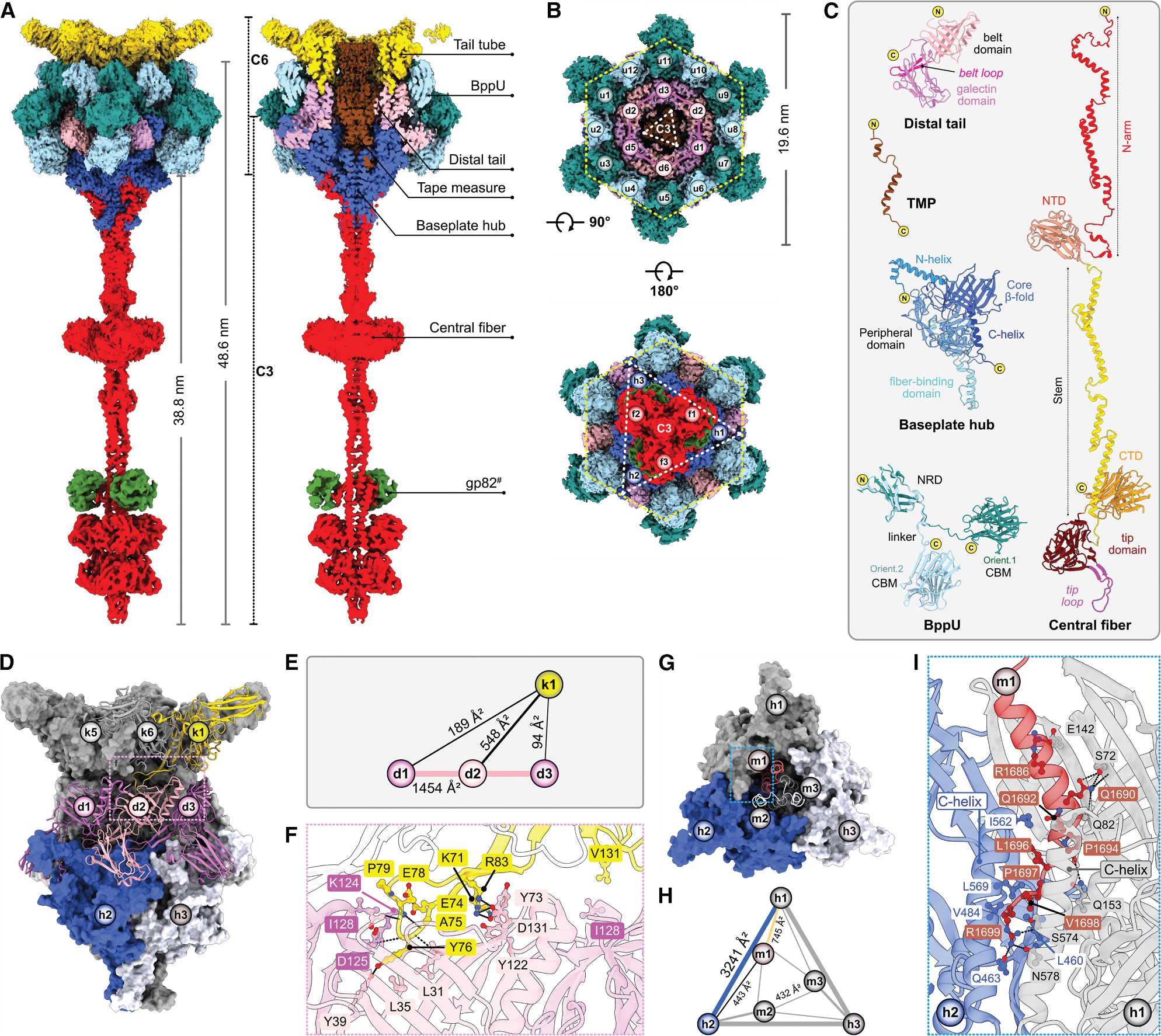
Structural overview of Douge baseplate-tail tip complex (A) Composite cryo-EM map of the Douge baseplate, with a z-clipped view showing the arrangement of its protein components. (B) Top and bottom views display the positioning of various proteins within the baseplate, including TMP within the lumen of the hexameric distal tail-trimeric BHPs, along with trimeric CFPs, and the hexameric arrangement of dodecameric BppUs. (C) Atomic structure of individual protein subunits of the baseplate, displaying their structural domains. Two different orientations of CBM of BppU are shown and colored differently. (D) Overview of a TTP ring and core baseplate (hexameric DTP-trimeric BHP), highlighting the TTP-DTP interaction. The pink boxed area references (F). (E) Buried surface area between TTP and DTP at different interfaces is illustrated. (F) Detailed interactions between TTP and DTP are shown, with key interacting residues in ball-and-stick models. (G) Top view of TMP-BHP association is displayed. The blue boxed area references (I). (H) Buried surface areas between TMPs and the BHPs at different interfaces are indicated. (I) Detailed interactions of TMP C terminus with BHP, shown as ribbon diagrams with key interacting residues in ball-and-stick models. H-bonds are displayed in black dotted lines.

The baseplate, situated at the distal end of the tail tube, is the most structurally diverse sub-component and plays a crucial role in host recognition and genome ejection.^53^ The composite map of the baseplate shows an outer diameter of 19.6 nm and a length of 48.6 nm. The tail tip, formed by the CFP alone, is 38.8 nm long. The baseplate consists of multiple copies of six proteins: three TMP, six DTP, three BHP, twelve BppU, and three CFP subunits, along with three subunits of a hypothetical protein component, likely gp82 (Figures 5A–5C), which requires further validation.

The DTP ring attaches to the distal end of the tail tube, with each subunit displaying two domains: an N-terminal belt domain (1–142) and a C-terminal galectin domain (143–291).^13^ The belt domain adopts a twisted β sandwich structure featuring a central helix and belt loop, while the galectin domain extends outward with a β-barrel-like fold (Figure 5C). The hexameric DTP ring attaches to the TTP ring via the belt domain, with interactions involving six H-bonds (Table S3) and several hydrophobic contacts, with a total buried surface area of ~4,986 Å^2^ (Figures 5D–5F). Trimeric BHPs, the central hub of the baseplate, connect to the DTP ring in a symmetry-mismatched arrangement via belt loop and galectin domains. This interaction is complex, including several H-bonds and a strong network of hydrophobic contacts (Figures S6A–S6C). The BHPs form a funnel-shaped trimer with five major domains: N-helix (1–41), core β-fold (42–147 and 487–560), peripheral domain (148–368), fiber-binding domain (369–486), and C-helix (561–587). The core baseplate, formed by the DTP and BHP rings, encircles a hexamer of homodimeric BppUs (Figure 5B), each composed of an N-terminal ring domain (NRD; 1–100) and a C-terminal carbohydrate-binding module (CBM; 116–311), connected by a flexible linker (101–115) (Figure 5C). BppU-NRDs form a dodecameric ring around the DTP belt domain, while the CBMs adopt two distinct orientations (Figure 5C) and form a hexamer of dimers that interface with the BHP peripheral domains (Figures S7A and S7B). DTP-BHP, DTP-BppU, and BHP-BppU associations follow C6-C3, C6-C12, and C3-C6 symmetry-mismatched junctures, respectively (Figures 5B, S7C, and S7D). Below the BHP, trimeric CFPs (forming a 38.8 nm fiber) anchor to the fiber-attachment domain via their N-arms through a strong network of H-bonds, hydrophobic contacts and π-π stacking interactions (Figures 5B and S6D). The CFP is composed of five domains: N-arm (1–164), N-terminal domain (NTD; 165–321), stem (322–484), tip domain (485–688), tip loop (502–529), and C-terminal domain (CTD; 689–878) (Figure 5C). Both the NTD and CTD share a similar structural fold to the CBM of BppU, suggesting the CFP functions as a receptor-binding protein (RBP).

The cryo-EM map of the baseplate hub reveals density (2.63–2.75 Å resolution) within the inner chamber (Figure 5A), which we assign to the C terminus of the TMP (residues 1,658–1,699). The structure reveals a trimeric arrangement within the baseplate lumen, suggesting that the 1,699-residue-long TMP forms a trimer throughout the tail tube channel. The TMP C termini insert into the DTP-BHP lumen via C3-C6 and C3-C3 symmetry interactions (Figure 5B). Detailed analyses reveal a strong H-bond network (Table S3) and hydrophobic contacts (Figures 5G–5I) between the TMP and BHP, burying a surface area of ~3,564 Å^2^ (Figure 5H). Additional electrostatic contacts (S1658^m3^–N34^d3^/Q36^d3^) are observed between the TMP and DTP (Figure S6E). The TMP density extends throughout the tail tube but becomes too diffuse (within the connector channel) to assign to specific protein residues. Previous studies indicate that the TMP directly interacts with genomic dsDNA, either within the tail tube in bacteriophage lambda^54^ or at the connector region near the terminator in DT57C.^36^ In Douge, the density corresponding to this region shows no clear discontinuity to distinguish the TMP from dsDNA density. Thus, the boundary is inferred near the stopper based on insights from phages Bxb1, SPP1, and JBD30.^16,34,55^

### Comparison of Douge structural components with other siphophages

To better understand structural similarities and differences between Douge and other siphophages, we compared their major sub-components. The Douge capsid exhibits dimeric CCPs located on the icosahedral plane, bridging C-hexon and P-hexon capsomeres. Arthrobacter phage Bridgette also features dimeric CCPs but positioned around the penton, bridging two P-hexon capsomeres.^14^ In contrast, *Escherichia coli* phage lambda and FCWL1, *H. pylori* phage KHP30, and *P. aeruginosa* phage JBD30 feature trimeric CCPs surrounding their capsomeres (Figure S8A).^55–58^ Despite the similar protein folds within the connector regions, the overall architecture varies significantly among siphophages (Figure S8B). For instance, the portal protein of Douge possesses a barrel structure that is absent in *B. subtilis* phage SPP1, lambda phage, JBD30, and *E. coli* phage DT57C but present in *Shigella flexneri* phage Sf6, a podophage.^22,36,54,55^ Additionally, DT57C lacks the stopper ring,^36^ and SPP1 exhibits a narrow connector channel of ~12 Å diameter and a genome gating mechanism, while Douge, lambda, JBD30, and DT57C all have a wider channel (>21 Å) (Figure S8B).

Although all TTPs share a common β-sandwich fold and β-hairpin loop enabling hexameric/trimeric assembly (Figure S9A), structural variations exist. For instance, Ig-like domains are found in Douge, JBD30, and *E. coli* phage T5, whereas SPP1 exhibits a distinct C-arm.^40,55,59^ Comparison of baseplate regions between Douge, T5, and lambda highlights this as the most structurally diverse component.^60,61^ In Douge, the DTP is surrounded by 12 BppU protomers that are absent in both T5 and lambda phages. T5 instead includes two additional proteins (a minor tail protein and an L-shaped tail fiber protein) between the TTP and DTP. Baseplate hub architecture also differs: Douge and T5 contain trimeric BHPs, while lambda shows three distinct proteins: BHP, BHP-CFP, and TAP (tail tip assembly protein) (Figure S9B). The central fiber structure further diverges both in length and domain organization, emphasizing their structural diversity.

### Comparison between genome-packed and genome-free mycobacteriophages

In addition to the cryo-EM structure of genome-packed Douge, we reconstructed a partial cryo-EM structure of genome-free Douge, including the capsid, connector, and tail tube. Resolutions achieved for the capsid, connector, and tail tube maps were 3.58, 3.77, and 3.16 Å, respectively (Figures S1A–S1E, S2D, and S2E). Local refinement improved the capsid and connector resolutions to 3.12 and 3.53 Å, respectively (Figures S1C and S1E).

To infer the mechanism of genome release, we compared the structures of genome-packed and genome-free virion components. In genome-free capsid, we observed no CCPs are attached onto the capsid shell (Figure S10A). A 5.3 Å expansion at the 3-fold axis was observed, suggesting that genome release likely induces detachment of the CCPs from the capsid shell (Figure S10B), leading to capsid relaxation. Connectors in both the genome-packed and genome-free states retained an open conformation, with the narrowest channel diameter of 21.4 Å, and their overall structure remains nearly identical (root-mean-square deviation [RMSD]: 0.63 Å; Figures S10C and S10D). Two differences were observed: (1) the densities corresponding to TMP and dsDNA within the connector channel were absent in the genome-free state and (2) the C termini of the portal (residues 508–545) in the genome-free connector became unresolved (Figure S10C), forming a blob-like density similar to that observed in Bxb1 after genome release.^16^

Previous cryo-EM studies of bacteriophage T5 and lambda reported substantial conformational changes in baseplates upon receptor binding.^60,61^ Cryo-ET analysis of mycobacteriophage Bxb1 similarly suggests that the baseplate opens during host interactions.^16^ However, low-resolution structural studies of T5 and Bxb1 indicate that the dimensions and torsion of the tail tube remain unchanged during genome ejection.^16,39^ Consistent with this, our genome-free structure (likely not induced by receptor binding) showed no structural changes between the tail tubes (RMSD: 0.3 Å; Figures S10E and S10F). These findings indicate that a mechanical transmission signal from the baseplate to the portal via the tail tube during phage-host interaction seems unlikely. Given the extensive interactions of the TMP with the BHP, we propose that the TMP functions as a physical seal for genome ejection and that the genome release is triggered solely by baseplate opening.

In most genome-free phages, the baseplate was not observed (Figure S10G). Only a small fraction (fewer than 50 in ~6,000 genome-free phages), representing stages during and after genome ejection, can be clearly observed with the crown-shaped baseplate, indicating the open conformation (Figure S10H). In contrast, genome-packed phages consistently exhibited a diamond-shaped baseplate with a long tip, indicative of a closed state (Figure S10I). These observations support that baseplate opening initiates TMP release, leading to genome ejection and subsequent loss of the baseplate.

### Douge is covered with glycan-binding domains

**Figure 6.**
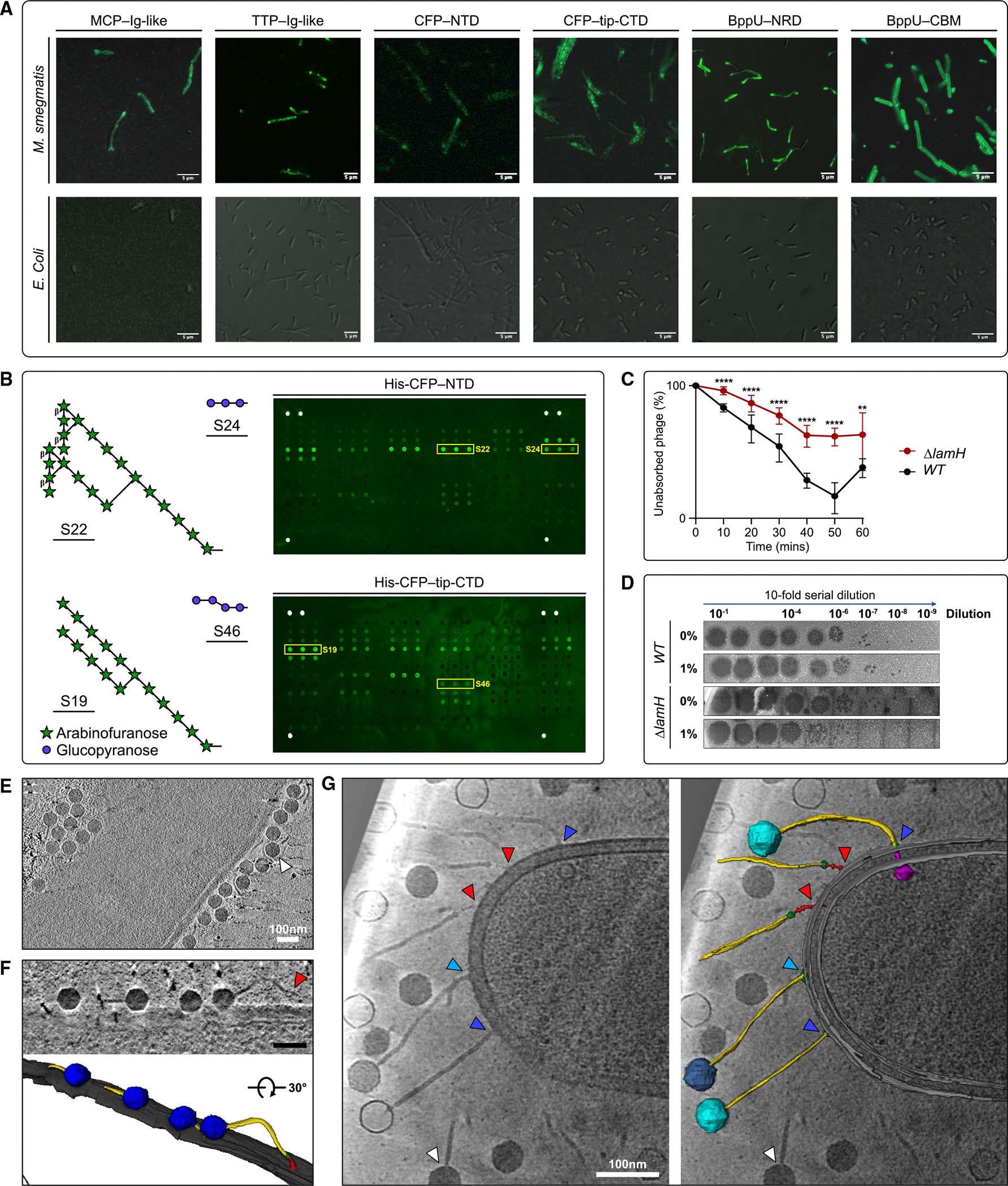
Douge-host interaction (A) Confocal microscopy images showing the surface interaction of *M. smegmatis* and *E. coli* with various Douge glycan-binding modules labeled by green fluorescent dyes. Scale bar: 5 μm. (B) Representative glycan array results demonstrating the binding of His-CFP-NTD and His-CFP-tip-CTD to components of the mycobacterial glycan array. Glycans were printed in triplicate; the green fluorescence indicates protein binding. Representative LAM and α-glucan fragments and their associated fluorescence intensities are shown to the left of the array. Glycans that are not structurally related to LAM/AM are present on the array and do not bind, thereby serving as negative controls (see Table S4 for additional data and a list of glycan structures). (C) Adsorption profile of Douge phage to *M. smegmatis* WT and *ΔlamH* mutant strains. The remaining percentage of free phage (*y* axis, log scale) is plotted over time. Data represent mean ± SEM at each time point as indicated. Statistical significance was assessed through t test. **p < 0.01 and ****p < 0.0001. (D) Mycobacteriophage Douge forms plaques with similar efficiency on *M. smegmatis* WT and *ΔlamH* mutant strains at dilutions up to 10^−8^. In the presence of 1% maltodextrin, Douge efficiency of plating (E.O.P.) remains changed on the WT strain but drops 10-fold on the *ΔlamH* mutant. Effects at lower maltodextrin concentration are shown in Figure S11C. (E) Representative cryo-ET tomographs of parallel phages. (F) Additional cryo-ET tomograph and segmentation of four parallel phages; one phage shows baseplate contact with the host cell wall. (G) Cryo-ET tomographs and the corresponding segmentations showing free Douge phage (white arrow) and phages at infection stage I (red arrows), stage V (blue arrow), and stage VI (cyan arrows). A hollow ball-like density attached to the cytosolic side of the inner membrane is highlighted in magenta. Scale bars in (E)–(G): 100 nm.

Amino acid sequence and structural analyses confirmed that the MCP and TTP subunits possess Ig-like domains, hypothesized to mediate weak carbohydrate interactions and contribute to host specificity.^62,63^ In an assembled Douge structure, 919 such domains project away from the phage surface and could conceivably interact with the bacterial host. The mycobacterial cell wall is fundamentally different from those of gram-positive and gram-negative (e.g., *E. coli*) bacteria,^64^ and its glycans are similarly distinct.^42^ In mycobacteria, the cell wall is covered with a variety of unusual glycans, predominantly LAM and its delipidated form, AM.^42^ To investigate whether the Ig-like domains of the MCP and TTP specifically recognize mycobacterial surface glycans, individual Ig-like domains were recombinantly expressed, purified, and chemically labeled with Cy3 fluorescent dye. Confocal microscopy confirmed that these Ig-like domains bind to the cell surface of *M. smegmatis* but not *E. coli*, indicating specificity (Figures 6A, left two images, and S11).

We further reasoned that other surface-exposed protein domains may also bind to mycobacterial glycans. A structure similarity search using the Dali server^65^ revealed that CFP-NTD and CFP-tip-CTD, as well as BppU-NRD and BppU-CBM, share similar structural folds with various carbohydrate-binding proteins (Table S2). Accordingly, we expressed and Cy3 labeled each domain and found that they all bind to the surface of *M. smegmatis* but not *E. coli* (Figures 6A and S9), supporting their potential roles as RBPs.

To better understand their host surface glycan specificity, the recombinant proteins were screened against an array of structurally defined fragments of various classes of mycobacterial cell wall glycans.^66^ This resource has previously been used to characterize the binding of a number of proteins, including lectins and monoclonal antibodies,^66,67^ to these glycans. Using this approach, all six surface-exposed proteins, MCP-Ig-like, TPP-Ig-like, CFP-NTD, CFP-tip-CTD, BppU-NRD, and BppU-CBM, bound to fragments of major surface antigens of mycobacteria, LAM/AM and α-glucan. Shown in Figure 6B are representative images of these array binding experiments using Cy3-labeled CFP-NTD (top) and CFP-tip-CTD (bottom). Both bind to capsular α-glucans and LAM/AM fragments, particularly extended branched chains containing arabinofuranose residues (see Table S4 for fluorescence intensity data). Similar binding profiles were observed for MCP-Ig-like, TPP-Ig-like, BppU-NRD, and BppU-CBM.

To assess the importance of LAM/AM in Douge-*M. smegmatis* interactions, we took advantage of a recently reported *M. smegmatis* mutant lacking the LamH glycoside hydrolase.^68^ This enzyme liberates AM from its lipid-linked counterpart, LAM. Consequently, deletion of *lamH* leads to the retention of LAM in the cytoplasmic membrane and the absence of AM in the cell wall periphery and capsule. We compared the Douge adsorption efficiency between wild-type (WT) and *ΔlamH M. smegmatis* strains. Approximately 32% of Douge adsorbed to the WT strain after a 10 min incubation, increasing to ~85% after 50 min. In contrast, only ~9% and ~58% of Douge adsorbed to the *ΔlamH* mutant strain within the same time frame, showing statistically significant slower adsorption (Figure 6C). These results suggest that surface AM is important for efficient Douge attachment, which we hypothesize results from interactions with the surface-exposed proteins (i.e., MCP, TPP, CFP, and BppU) on the mycobacteriophage surface. Despite the reduction in adsorption, Douge exhibits similar infectivity in both the WT and *ΔlamH* mutant strains, with an efficiency of plating (E.O.P.) of 1. We propose that this arises from the ability of surface-exposed proteins to also bind to another capsular component, α-glucan (Figure 6B; Table S4). To test this, we pre-mixed Douge with 1% maltodextrin (DP10–40), which structurally resembles mycobacterial α-glucan. We hypothesized that, under these conditions, the added maltodextrin would bind to surface-exposed proteins, thereby preventing interactions with the host. Consistent with this, Douge infectivity toward the *ΔlamH* mutant strain was significantly reduced under these conditions, with an E.O.P. of 0.1 (Figure 6D). This reduction was concentration dependent and observed only in the *ΔlamH* strain, not in the WT, suggesting a synergistic effect between the binding of α-glucan and capsular AM on Douge infectivity (Figure S11C).

### Phage-host interaction

To investigate Douge-*M. smegmatis* interactions, we used cryo-ET, which allowed *in situ* observation at a near-native state with nanometer resolution. We observed the outer membrane and plasma membrane as distinct dense layers, indicating an *M. smegmatis* cell wall thickness of ~28 nm (Figures 6E–6G), consistent with previous work.^69^ Analyses of 22 tomograms revealed 576 phage particles, of which about 11% (65 phages) directly interacted with the bacterium.

**Figure 7.**
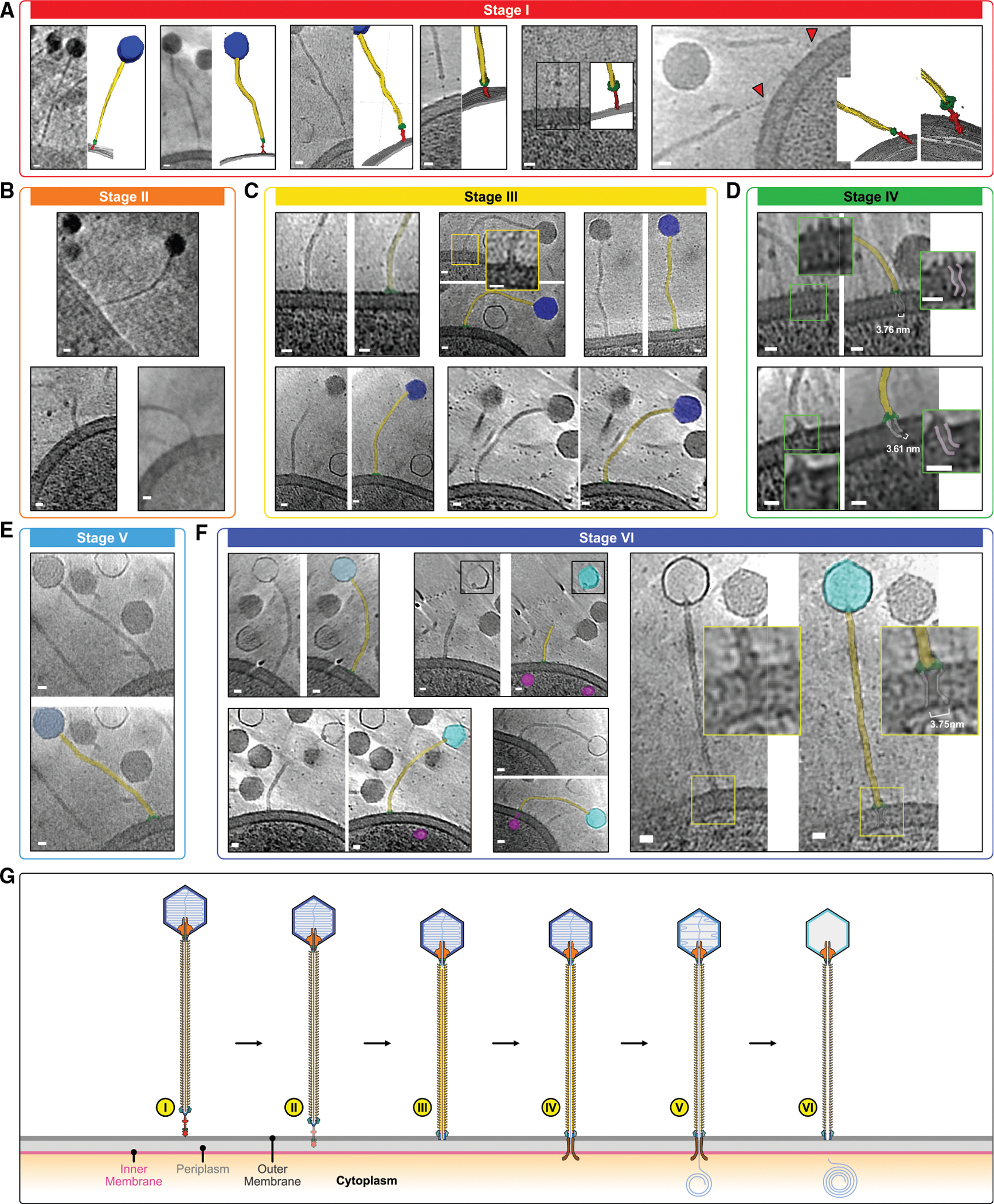
23 cryoelectron tomographs of Douge-host interaction and proposed genome delivery mechanism (A) Stage I: initial attachment of central fiber protein to the host surface. (B) Stage II: insertion of central fiber into the outer membrane. (C) Stage III: attachment of baseplate onto the host surface. (D) Stage IV: formation of a channel-like structure, with the boundaries indicated by white lines. (E) Stage V: capsid and tail tube lumen show less density, indicating the ongoing process of genome ejection. (F) Stage VI: capsid and tail tube lumen show no density, indicating the end of genome ejection. (A–F) For each stage, raw tomograms (left) and segmentations highlighting key structural components (right) are shown. Capsids, tail, baseplate hub, and central fiber proteins are colored in blue, green, red, and yellow, respectively. Zoom-in views of the highlighted regions are shown in the colored boxes. Scale bars: 20 nm. The annotations in (C), (D), and (F) are based on densities observed in a 2D tomographic slice (average thickness: 10.92 nm), with highlighted regions containing features of interest. (G) Schematic illustration summarizing the proposed six-staged mechanism of Douge phage attachment, host-membrane penetration, and genome delivery.

Two distinct phage-mycobacterium interactions were identified based on phage orientation in relation to the host cell wall, “parallel” and “standing” (Figures 6E–6G; Video S1), resembling observations from the podophage P-SSP7.^70^ In the parallel orientation, we observed 42 phages, where primarily the capsid appears close to the cell wall, and the flexible tail tube adopts a range of orientations (Figure 6F). In the standing orientation, 23 phages were observed, where either the baseplate or the central fiber (tail tip) attaches to the cell wall (Figures 6G and 7A–7F). Based on the contact point and capsid density, we grouped the standing phages into several categories (Figures 7A–7F) and postulate that these correspond to different stages of infection. In stage I, the central fiber remains observable with strong density at the contact point on the bacterial surface, which we propose is irreversible adsorption to the host surface to initiate infection (Figure 7A).^71^ In stage II, the penetration of the central fiber (tail tip) through the outer membrane was observed (Figure 7B). Several stages (III–VI) can be observed in which the CFP disappears, and the core baseplate region (DTP-BHP-BppU) is attached to the outer membrane. Stages I–III retain the density-intact genomic dsDNA (Figure 7C). During stage IV, the formation of an ~3.7 nm hole at the junction of the baseplate and outer cell wall is seen, accompanied by the creation of a channel-like structure in the periplasmic region (Figure 7D). Previous studies have suggested that trimeric TMPs, which contain two transmembrane regions, are released to form a channel spanning the bacterial envelope, preventing genome degradation by periplasmic endonucleases.^72–74^ Therefore, we propose that the channel-like structure observed in the cryo-ET images arises from trimeric TMPs. In stage V, one standing phage exhibited weak density inside the capsid chamber, which we postulate is the phage undergoing the genome ejection process (Figure 7E). In stage VI, the baseplate remains attached, but the tail tube channel and capsid chamber are empty, suggesting genome ejection (Figure 7F). While this proposed Douge-host infection model is speculative, constructed from static cryo-ET images, Figure 7G outlines a possible timeline of events. Although further validation is needed, we nevertheless believe this model provides an intriguing possible infection pathway that can be tested in subsequent investigations.

## DISCUSSION

Although over 14,400 mycobacteriophages have been isolated, the work reported here represents one of two atomic-resolution structures of these viruses, including the recently reported structure of Bxb1.^16^ Our comprehensive cryo-EM investigation of Douge reveals that 1,105 protein building blocks are required to form this 400 nm siphophage. These proteins, belonging to only 13 protein subunit families, are self-assembled into different symmetrical subcomplexes, including those with symmetry-mismatched interfaces. Our composite map allows us to elucidate the critical protein interaction networks between individual subcomplexes that are necessary to assemble the entire siphophage.

In bacteriophages, several types of CCPs, also referred to as decoration/auxiliary proteins, have been previously reported.^14,57,75–77^ In Douge, we identify a distinct type of CCP, characterized by the shortest protein length and a unique structure, distinct from those reported previously (Figure S8A), that forms a dimer located at the C-hexon-P-hexon interface on the icosahedral plane. In addition, our cryo-EM map revealed that TMPs form a homotrimer and intensively interact with BHPs, providing structural insights to support a model proposed previously for bacteriophage T5.^60^ We propose that following BHP opening, the expulsion of TMPs from the tail tube leads to genome release.

Unlike structures reported for SPP1 and DT57C,^34–36,78^ we did not observe an obvious gating system in the connector region. The narrowest diameter of the connector channel in Douge is 21.4 Å, similar to that reported for phage lambda,^54^ and it is wide enough to allow dsDNA to pass through. We further observed that the connector and tail tube remain the same in both genome-packed and genome-free states, suggesting that no mechanistic transmission occurs via the tail tube upon phage-host interaction and that there is no DNA gate-opening mechanism by the stopper ring. Instead, we observed that dsDNA and TMP occupy the lumen of the connector region. Unlike myophages, which rely on tail tube contraction to induce conformational changes in the connector region for genome ejection, we hypothesize that in Douge, conformation changes in the baseplate region upon host interaction facilitate the release of the TMPs, which, in turn, triggers genome ejection. The process is driven by internal pressure stored within the capsid, providing the necessary force for TMP release and genome ejection.^79^

Previous studies suggested that TMPs create a channel spanning the bacterial cell wall to prevent viral genome degradation.^73,80,81^ Our cryo-ET images reveal a distinctive channel-like structure extending from the distal end of the baseplate, originating at the outer membrane, passing through the periplasm, and reaching the inner membrane (Figures 7D and 7F). Additionally, a curved-density, possibly phage dsDNA is observed on the cytosolic side of the inner membrane (Figures 6G and 7F; Video S1).

Around 25% of tailed phages, including Douge, possess Ig-like domains on their surface.^62,63^ We demonstrate that the Ig-like domains on the Douge capsid and tail tube label the mycobacterial surface, which we propose facilitates phage attachment in a parallel orientation, as shown in cryo-ET images (Figures 6E and 6F). Additionally, the baseplate appears capable of projecting in various directions due to the flexible tail tube (Figure 6F). Given the natural aggregation tendency of mycobacteria,^82^ this flexibility may facilitate baseplate attachment to neighboring cells when the capsid is attached to another in a parallel orientation. Alternatively, the flexible tail tube may increase the likelihood of baseplate contact with the same host cell while maintaining a parallel orientation. The latter possibility was probed in molecular dynamics (MD) simulations, which revealed transitional interactions between the capsid and host cell surface over time (Figure S12), assuming weak binding of MCP-Ig-like domains with the surface glycans. MD analysis also revealed that the flexible tail increases the possibility of baseplate-cell surface contact from 32% to 76% (Figures S12B–S12D). Moreover, the phages with rigid tails diffuse further away from the host (distance: 464.90 ± 104.34 nm; Figure S12E; Video S2) compared to phages with flexible tails (124.76 ± 93.31 nm) (Figure S12E; Video S3). While these findings support the role of the flexible tail in facilitating baseplate attachment, further validation is required.

In summary, we present the molecular architecture of the entire siphophage Douge at atomic resolution, revealing crucial interactions that allow the structure to form from multiple copies of only 13 protein subunits. Our *in situ* cryo-ET work reveals several key snapshots of phage-mycobacterium interactions that we propose may be important during mycobacteriophage infection (Figure 7G). Notably, we identified six glycan-binding domains on the MCP, TTP, BppU, and CFP that specifically recognize the *M. smegmatis* cell surface glycan, suggesting their function as RBPs. This finding was further supported by demonstrating that these proteins bind to fragments of mycobacterial LAM and that Douge adsorbs more slowly to an *M. smegmatis* strain lacking capsular AM than to the WT strain. These results open up the possibility that altering the carbohydrate-binding specificities of these proteins by structure-based phage engineering may give rise to mycobacteriophages that target other hosts. For example, inserting domains that can bind to mycobacterial glycopeptidolipids may allow them to infect *Mycobacterium abscessus*, the surface of which is characterized by such molecules.^83^ Similar approaches could be used to target other mycobacteria that produce unique glycans. Taken together, our findings provide an essential foundation upon which further mechanistic and functional aspects of siphophage-mycobacteria interactions can be explored.

### Limitations of the study

While this study offers high-resolution structural insights into the mycobacteriophage Douge, some limitations remain. The mechanism of genome ejection, inferred from comparisons of genome-packed and genome-free structures, requires further investigation. The density within the connector needs to be better resolved; it is currently difficult to determine whether it corresponds to dsDNA or the TMP. Additionally, due to the dynamic nature and relatively low resolution of the tail tip, the identity and function of the hypothetical protein (likely gp82) associated with the central fiber remain speculative and require experimental validation. Moreover, the current description of the genome ejection process is primarily based on nanometer-resolution cryo-ET; higher-resolution studies are necessary to elucidate the detailed structural and mechanistic aspects of this process in Douge. Despite these limitations, the high-resolution structure of Douge and its demonstrated binding preferences for LAM/AM fragments advance our understanding of mycobacteriophage biology and provide a foundation for future phage engineering efforts.

## RESOURCE AVAILABILITY

### Lead contact

Further information and requests for resources and reagents should be directed to and will be fulfilled by the lead contact, Meng-Chiao Ho (joeho@gate.sinica.edu.tw).

### Materials availability

This study did not generate new, unique reagents.

### Data and code availability

- Cryo-EM reconstructions and protein models have been deposited at EMDB and PDB, respectively, and the accession numbers are listed in the key resources table.
- The composited map of the 2x binned cryo-EM map of the complete Douge, along with 27 PDB coordinate files, is available on Figshare (https://doi.org/10.6084/m9.figshare.26494552).
- This paper does not report original code.
- Any additional information required to reanalyze the data presented in this paper is available from the lead contact upon request.

## STAR★METHODS

### EXPERIMENTAL MODEL AND STUDY PARTICIPANT DETAILS

Mycobacteriophage Douge was isolated from soil samples collected in Hsinchu, Taiwan. The study used *M. smegmatis* strains mc^2^155 and Δ*lamH*, and *E. coli* strains BL21(DE3) and DH5α. *M. smegmatis* mc^2^155 strain was specifically used for the isolation of phage Douge.

### METHOD DETAILS

#### Host strain and growth media

*Mycobacterium smegmatis* mc^2^155 was used as the host for mycobacteriophage isolation and amplification. It was grown in Middlebrook 7H10 agar (HiMedia, India) with 0.55% (*v/v*) glycerol, 50 μg/mL carbenicillin (CB) (Sigma-Aldrich, USA) and 10 μg/mL cycloheximide (CHX) (Acros Organics, USA). For liquid culture, it was grown in Middlebrook 7H9 broth (BD Difco, USA) supplemented with 40% (*v/v*) glycerol and containing 1 mM CaCl_2_ (Acros Organics, USA), 50 μg/mL CB, 10 μg/mL CHX and 10% (*v/v*) albumin dextrose complex (ADC). ADC is composed of 0.53% (*w/v*) Bovine Serum Albumin (Roche, Switzerland), 0.9% (*w/v*) NaCl and 2.1% (*w/v*) Dextrose (Fisher Scientific, USA). Double-layer agar method was used for phage isolation and amplification. To enrich the mycobacterial growth, the bottom agar was prepared with 2.16% (*w/v*) Middlebrook 7H10 agar, 0.55% (*v/v*) glycerol, 50 μg/mL carbenicillin and 10 μg/μL cycloheximide. To visualize the phage growth, the soft top agar was mixed with Middlebrook 7H9 and MBTA (0.47% (*w/v*) Middlebrook 7H9 broth, 0.7% (*w/v*) agar) (1:1, *v/v*), 1 mM CaCl_2_, 50 μg/mL carbenicillin, 10 μg/mL cycloheximide, the mycobacterial host (OD_600_ 1.5–2.0), and the phage sample. The mixture was then poured into a plate containing the bottom agar.

#### Phage isolation

For phage isolation, a damp, warm, aerated soil sample with decaying matter was collected in Hsinchu country. The isolation method was described in Actinobacteriophage Database with minor modification.^43^ The soil sample was mixed vigorously with 10 mL phage buffer (10 mM Tris, 10 mM MgSO_4_, 70 mM NaCl, and 1 mM CaCl_2_ at pH 7.5) and 5 mL of supernatant was filter sterilized using a 0.22 μm filter. To increase the sensitivity of phage detection, sample supernatant was mixed with 7H9 medium and *M. smegmatis* (OD_600_ 0.6), and incubated at 37°C for 16 hours. Purification was conducted using T-streaking method to make sure the phages were from the identical genetic clone. Additional details on Douge can be found at https://phagesdb.org/phages/Douge/.

#### Phage amplification

The phage amplification was described previously with minor modification.^104^ A small-scale amplification was conducted first to obtain enough phage particles for large-scale amplification and storage. After amplification, phage particles were pelleted by mixing the lysate with 1 M NaCl and 10% (*w/v*) polyethylene glycol (average molecular weight 8,000) (Sigma-Aldrich, USA) and centrifuged at 6,400 rpm (HIGHConic III Fixed Angle Rotor, ThermoFisher Scientific, USA) for 10 minutes. After pellet resuspension, phages were concentrated by cesium chloride (CsCl) (ThermoFisher Scientific, USA) ultracentrifugation using Type 50.2 Ti Fixed-Angle Rotor (Beckman) at 38,000 rpm for 16 hours at 4°C to obtain high titer phage lysate. The phage titers were tested and recorded using spot test described before.^104^ The high titer phage stock in CsCl was stored in 4°C for long term storage.

#### Genomic DNA extraction

Phage DNA samples were extracted from high-titer CsCl phage stocks following the phenol–chloroform–isoamyl alcohol (PCI) protocol described previously.^104^ High-titer CsCl phage stock was dialyzed against 1 L phage buffer without CaCl_2_ at 4°C overnight and then an extra 2 hours with 1 L fresh phage buffer. The DNA concentration was measured using a Nanodrop One^c^ (ThermoFisher Scientific, USA) after DNA extraction.

#### Genomic DNA sequencing, assembly, and annotation

Phage genome samples were sequenced by Academia Sinica High Throughput Sequencing Core at the Biodiversity Research Center using MiSeq Reagent Kit v2 Micro PE150 (Illumina, USA) in Illumina MiSeq v2 sequencing mode with 300 cycles (pair-end 150 bp). Sequenced raw reads were assembled using GS *De Novo* Assembler (Newbler) v2.9 (Roche/454 Life Sciences, Branford, CT). Genome annotation was checked manually using DNA Master v 5.23.6 (http://cobamide2.bio.pitt.edu), and gene functions were generated automatically using the BLASTp on DNA Master and assigned manually by using PhagesDB BLASTp,^85^ HHpred,^86^ and the PECCAN (http://pecaan.kbrinsgd.org). Transfer ribonucleic acids (tRNAs) were predicted automatically by ARAGORN v1.1^87^ on DNA Master and examined manually by ARAGORN v1.2.38 and tRNAscan-SE v2.0.^88^ Complete phage genomes were deposited and updated on both PhagesDB (https://phagesdb.org/phages/Douge/) and Phamerator.^89^

#### Cryo-EM sample preparation

Cryo-samples were prepared using Thermo Scientific Vitrobot (Mark IV) at 4°C with 100% humidity. 4 μl of purified phage was applied onto glow-discharged Quantifoil holey carbon grids coated with (or without) a thin layer (~2 nm) of carbon (Quantifoil GmbH, Germany). Following a brief incubation of 10 seconds, the excess solution was blotted away the grid was plunged into liquid nitrogen-cooled liquid ethane.

#### Cryo-EM imaging

The data acquisition was automated using EPU-2.7.0 software (Thermo Fisher Scientific) on a 300 kV Titan Krios microscope (Thermo Fisher Scientific), equipped with a K3 Summit detector and Bio-Quantum energy filters (Gatan), operating in super-resolution mode. The slit width of the energy filters was adjusted to 15 eV. Raw movie stacks were captured at a magnification of 81,000×, corresponding to a pixel size of 0.5305 Å/pixel with e defocus range between −1.0 to −2.2 μm. Each movie consisted of fifty frames with a total dose of approximately 50 e-/Å^2^. Detailed parameters for cryo-EM imaging are provided in Table S1.

#### Cryo-EM data processing

All super-resolution movies were motion corrected and dose weighted using MotionCor2^90^ with two-fold binning, resulting in a pixel size of 1.061 Å/pixel. The contrast transfer function (CTF) information was then estimated by CTFFIND4.1.^91^ Details of the data processing procedures and cryo-EM reconstruction are provided in Figures S1 and S2. Statistical information regarding the cryo-EM reconstructions, focus-refined density maps, and model validations can be found in Table S1.

For the Douge capsid, a small subset of particles was picked followed by 2D classification in cryoSPARC v4.1.^92^ The selected 2D classes were then used as templates for particle picking across the entire dataset. Simultaneously, the particles from the small dataset were used to generate initial 3D models using ab-initio reconstruction with icosahedral (I) symmetry. A total of 3,026,432 particles were picked from three datasets and extracted with a box size of 860 pixels, which was rescaled to 128 pixels for subsequent 2D classification to remove poor-quality particles. The particles (263,946 particles combined from three datasets) corresponding to the capsid region of Douge, as identified from selected 2D class averages (Figure S1A), were further classified into four 3D classes using heterogeneous refinement with icosahedral (I) symmetry. Following additional iterations of 2D classification and heterogeneous refinement (3 classes, I symmetry), the high-quality particles representing the genome–packed capsid (94,629 particles) and genome–free capsid (21,074 particles) were re-extracted with a box size of 860 pixels and subjected to homogeneous refinement with icosahedral (I) symmetry. The cryo-EM structures of both genome–packed and genome–free capsids were obtained, achieving resolutions of 3.20 Å and 3.58 Å (Figures S1A–S1C), respectively. To improve the resolution of the MCP subunits, we conducted focused refinement to refine the densities of the Penton, P-hexon, and C-hexon capsomere regions. First, we expanded the symmetry (I symmetry) and then performed local refinement using a soft mask (Figure S1A) focusing on these specific regions without imposing the symmetry (C1). The resulting focused-refined maps exhibited improved features compared to standard icosahedral reconstructions and the resolutions were also enhanced as compared to the original reconstructions. The resolutions of the focus-refined maps for the genome–packed capsid were improved to 2.95 Å, 2.83 Å, and 2.80 Å for the penton, P-hexon, and C-hexon capsomere regions, respectively (Figure S1B). Similarly, the resolutions of the focus-refined maps for the genome–free capsid were improved to 3.37 Å, 3.16 Å, and 3.12 Å for the penton, P-hexon, and C-hexon capsomere regions, respectively (Figure S1C). It is worth to mention that the genome–free Douge was purified from the same band of CsCl gradient sedimentation as its genome–packed counterpart. Therefore, the genome–free Douge was likely to be initially packed with dsDNA and well assembled, but that genome ejection occurs before or during the cryo-EM sample preparation.

For the genome–packed connector, we started by removing the capsid protein density from the capsid map at 3.2 Å (Figure S1D) through particle subtraction, followed by symmetry expansion (I symmetry) and a mask application at the fivefold vertices for 3D classification (6 classes, C1 symmetry). About 13% of the particles showed density corresponding to the connector. These particles underwent 2D classification, and those within selected 2D classes were re-extracted (box size of 860 pixels, rescaled to 220 pixels) and subjected to ‘homogeneous reconstruction only’ to get an initial map of the connector. Next, we eliminated part of the capsid density from the remaining particles and re-centered the connector using template-based homogeneous refinement (3 iterations, C5 symmetry). After that, the particles (70,372 particles) were extracted to a box size of 512 pixels. Subsequently, the heterogeneous refinement (2 classes, C5 symmetry) was carried out to remove particles lacking connector density. The remaining particles underwent further homogeneous refinement with C5 symmetry to obtain the densities for the capsid region. A mask was applied to the portal region for 3D classification (6 classes, C1 symmetry) using C5 symmetry-expanded particles. One of the classes lacked the portal, while another class showed clear densities for both the portal (C12) and capsid (C5). Using this map as a reference, we aligned other maps to it, removed duplicate particles, and performed local refinement (C1), achieving a resolution of 3.74Å. To further improve the resolution in each region, we conducted additional non-uniform refinement with C6 and C5 symmetries to obtain the densities for the connector and capsid–vertex regions, respectively. The cryo-EM structures of the genome–packed connector at the portal region (C6 symmetry) and capsid region (C5 symmetry) were obtained, achieving resolutions of 3.18 Å and 3.44 Å (Figures S1D and S1E), respectively. To enhance the resolution of the different connector regions, we performed focused refinement using a soft mask focusing on the densities of the portal–adaptor (gp5 and gp9), stopper–terminator–tail tube (gp10, gp12, and gp13), and capsid–vertex (gp8) regions with C12, C6, and C5 symmetry, respectively. The resulting focused-refined maps displayed improved features compared to the original homogeneous refinement. The resolutions of the focus-refined maps for the connector were improved to 2.82 Å, 2.96 Å, and 3.25 Å for the portal–adaptor, stopper–terminator–tail tube, and capsid–vertex regions, respectively (Figure S1E). A composite map of genome–packed connector was created by fitting the high-resolution densities into the C1 connector map, which showed clear densities for both the portal (C12) and capsid (C5).

For the genome–free connector, we began by removing the capsid protein density from the genome–free capsid map at 3.58 Å (Figure S1D) using particle subtraction. Then, we conducted ab-initio reconstruction (3 classes, C1 symmetry) to obtain the initial map. Following heterogeneous refinement, approximately 60.4% of the particles remained. After an additional round of 2D classification, we proceeded with homogeneous refinement to achieve a structure at 4.15 Å. These particles were subsequently re-extracted (box size of 860 pixels, rescaled to 220 pixels). Next, we re-centered the connector using template-based homogeneous refinement (3 iterations, C6 symmetry). The particles were then extracted to a box size of 512 pixels. Heterogeneous refinement (2 classes, C6 symmetry) was performed to remove particles lacking connector density. The remaining particles (63.1%) underwent non-uniform refinement and local refinement without symmetry to obtain a portal density with a resolution of 6.03Å. To further improve the resolution in each region, we performed non-uniform refinement with C6 and C5 symmetries to obtain the densities for the connector and capsid–vertex regions, respectively. The cryo-EM structures of the genome–free connector at the portal region (C6 symmetry) and capsid region (C5 symmetry) were obtained, achieving resolutions of 3.78 Å and 4.22 Å (Figures S1D and S1F), respectively. To improve the resolutions of different connector regions, focused refinement was conducted using a soft mask (Figure S1D) focusing on the densities of the portal–adaptor (gp5 and gp9), stopper–terminator–tail tube (gp10, gp12, and gp13), and capsid–vertex (gp8) regions with C12, C6, and C5 symmetry, respectively. The resulting focused-refined maps displayed improved features, and the resolutions were also enhanced. Specifically, the resolutions of the focus-refined maps for the connector were improved to 3.53 Å, 3.89 Å, and 4.0 Å for the portal–adaptor, stopper–terminator–tail tube, and capsid–vertex regions, respectively (Figure S1F).

For the tail tube, an initial small set of particles was manually picked for 2D classification. The selected 2D classes were then used as templates for particle picking across the entire dataset. A total of 4,886,787 particles were picked extracted with a box size of 768 pixels, which was rescaled to 384 pixels for subsequent 2D classification to remove poor-quality particles. The particles identified from selected 2D class averages (2,265,916 particles combined from two datasets) were further used for initial reconstruction (C1 symmetry) and classified into three 3D classes using heterogeneous refinement with C1 symmetry. About 70.2% of the particles (1,591,245 particles) with the genome–packed straight tail tube features were used for further homogeneous refinement (C6 symmetry) to achieve a map with a resolution of 2.23 Å (Figure S2A). After particle extraction with a box size of 768, and performing homogeneous refinement (C6 symmetry), the final reconstruction achieved a resolution of 2.18 Å (Figures S2B and S2C). Helical reconstruction was also performed to calculate the twist and rise of the tail tube. The resolution of the tail tube from helical reconstruction (C6 symmetry) was achieved at 2.14 Å (Figure S2B), with a twist of 16.6° and a rise of 41.797 Å.

For the genome–free tail tube, we aimed to compare its structure with that of the genome–packed tail tube. To do this, we manually selected particles from the micrographs. A total of 15,389 particles were chosen from 954 micrographs and extracted with a box size of 384 pixels. These particles underwent 2D classification to remove any poor-quality ones, resulting in 12,010 particles identified from selected 2D class averages (Figure S2D). The particles were then subjected to homogeneous refinement with C6 symmetry to achieve a map with a resolution of 3.07 Å (Figures S2D and S2E). Additionally, helical reconstruction was conducted to determine the twist and rise of the tail tube. The resolution of the genome–free tail tube from helical reconstruction (C6 symmetry) was found to be 3.16 Å (Figure S2D), with a twist of 16.703° and a rise of 41.737 Å.

For the baseplate, we started by manually selecting particles for initial 2D classification. Selected 2D classes served as templates to pick particles from the entire dataset. A total of 4,615,065 particles were selected from three datasets and extracted with a box size of 512 pixels, later resized to 128 pixels for additional 2D classification to remove low-quality particles. From the selected 2D class averages, 98,766 particles were identified and extracted with a box size of 512 pixels. Then, we conducted an ab-initio reconstruction (2 classes, C1 symmetry) to obtain the baseplate’s initial maps. Following heterogeneous refinement (2 classes, C3 symmetry), about 86.1% of the particles remained. Further 2D classification was performed to refine particle selection, yielding 78,927 good particles. After non-uniform refinement with C3 symmetry, we achieved a baseplate map with a resolution of 2.97 Å (Figures S2F and S2G). To enhance resolution in different regions of the baseplate, focused refinement was carried out using a soft mask focusing on the densities of the top (gp13), center (gp17 and gp23), and bottom (gp16, gp18, and gp20) regions with C6, C6, and C3 symmetry, respectively. The resulting focused-refined maps displayed improved, and the resolutions were also enhanced. Specifically, the resolutions of the focus-refined maps for the connector were improved to 2.71 Å, 2.75 Å, and 2.63 Å for the top, center, and bottom regions, respectively (Figure S2G).

For the tail tip (central fiber region), we manually chose a small number of particles for preliminary 2D classification. Selected 2D classes were then used as templates to pick particles across the entire dataset by template picker. A total of 10,107,735 particles were selected from three datasets and extracted with a box size of 512 pixels, which was later scaled down to 128 pixels for further 2D classification to filter out low-quality particles. Particles identified from good 2D class averages (6,807,101 particles combined from three datasets) were then used for ab-initio reconstruction (C1 symmetry) and classified into four 3D classes using heterogeneous refinement with C3 symmetry. About 27.4% of particles with good baseplate features underwent further heterogeneous refinement (four classes, C3 symmetry), while about 72.6% of particles with poor baseplate features also underwent heterogeneous refinement (four classes, C3 symmetry) to rescue any good particles. Subsequently, 68.8% and 19.4% of particles from good and rescued classes of heterogeneous refinement (C3 symmetry) were subjected to additional rounds of ab-initio reconstruction (C1 symmetry), heterogeneous refinement (C3 symmetry), and 2D classification to eliminate bad particles. Particles (139,838 particles) identified from good 2D class averages were further classified into three 3D classes using heterogeneous refinement with C3 symmetry. These particles were then re-extracted with a box size of 620 pixels and classified into two 3D classes by heterogeneous refinement (C3 symmetry). The final 31,169 particles from the good 3D classes underwent further refinement to achieve a resolution of 4.26 Å by non-uniform refinement with C3 symmetry (Figures S2H and S2I). To enhance the resolution of different densities in the central fiber region, focused refinement was conducted to refine the densities of the top and bottom regions. Initially, symmetry expansion (C3 symmetry) was applied, followed by local refinement using soft masks (Figure S2H) focused on these specific regions without imposing symmetry (C1). Although only the overall resolution of the focus-refined maps for the bottom part improved to 3.88 Å (Figures S2H and S2I), both resulting focus-refined maps exhibited improved features compared to non-uniform refinement. The resolutions of the focus-refined maps for the top and bottom regions of the tail-tip were estimated to be 4.48 Å and 3.88 Å (Figure S2I), respectively.

The combined maps from focus-refined maps for capsids (genome–packed and genome–free), connectors (genome–packed and genome–free), and baseplate–tip were generated in Chimera.^93^ Initially, the focus-refined maps were aligned with the consensus maps and then merged using the ‘vop max’ command. Map sharpening and resolution estimation were performed using cryoSPARC v4.1. The overall resolution was assessed using the Fourier Shell Correlation (FSC) = 0.143 criteria, and the local resolution was determined within cryoSPARC v4.1. The resulting 3D density maps were visualized using Chimera^93^ and ChimeraX.^94^

#### Structure determination and model building

The models of individual protein subunits were predicted using AlphaFold2^44^ and then docked into the corresponding cryo-EM densities using Chimera.^93^ The unfitted regions were manually adjusted using the COOT program,^95^ and then further refined using PHENIX^96^ real-space refinement module. The atomic models were validated by PHENIX and the validation statistics are tabulated (Table S1). As for the hypothetic protein component, the local resolution of this corresponding density was improved using DeepEMhancer,^97^ and manually screened all possible Douge protein sequences using the *de novo* modeling protocol, DeepMainmast.^98^ Based on model-to-density fitting, we speculate that this hypothetical protein is likely gp82, which needs further validation (Figure S2J).

#### Cryo-ET imaging and data processing

A phage-host mixture with 10-nm fiducial gold beads (Ted Pella) was applied onto a freshly glow-discharged Quantifoil R 2/2 200 Holey carbon grid (Quatifoil GmbH, Germany). The excess solution was blotted away at 25°C and 100% relative humidity and plunge-frozen into liquid ethane using Thermo Scientific Vitrobot (Mark IV). The data acquisition was carried out with a 200 kV Talos Arctica microscope with a Falcon III detector (Thermo Fisher Scientific) operating in linear mode, or a 300 kV Titan Krios microscope (Thermo Fisher Scientific) equipped with a K3 Summit detector camera (Gatan) and a Gatan energy filter (with a slit width set to 20 eV) operating in counting mode. The tilt-series parameters included a tilt range of −50° to +50°, magnification of 28000x in Talos Arctica (with a corresponding pixel size of 3.61 Å), 33000x in Titan Krios (with a corresponding pixel size of 2.73 Å), and a tilt increment of 2°. The target defocus was set to between −5 and −6 μm and the total accumulated dose was approximately 100–120 e−/Å2. All image acquisition was done using Tomography software (Thermo Fisher Scientific). Frames of each tilt image were motion corrected by MotionCor2.^90^ The average tilt-series images were then aligned and reconstructed by weighted back projection with a SIRT-like filter (10 iterations) into tomograms using the ETomo-interface of IMOD v4.9.12.^99^ For visualization, the reconstructed tomograms were binned by four and denoised by Topaz v0.2.5^100^ or filtered with MATLAB script tom_deconv.^101^ Tomograms were inspected using the IMOD slicer window, and segmentation (shown in Figures 6F, 6G, and 7A) was performed using Amira software 2022.1 (Thermo Fisher Scientific) with the ‘Membrane Enhancement Filter’ module,^102^ followed by manual refinement. For Figures 7C, 7D, and 7F, the annotations are drawn from the densities displayed in a 2D tomographic image (with an average thickness of 10.92 nm) to highlight areas containing features of interest. A series of tomogram sections used for Figure 6G is shown in the Video S1.

#### Cloning, expression, and purification of Douge putative RBPs

Douge MCP–Ig-like (His-GST tagged), TTP–Ig-like (His-MBP tagged), CFP–NTD (His-tagged), CPF–tip-CTD (His-tagged), BppU–NRD (His-tagged), and BppU–CBM (His-tagged) regions were cloned into modified pET vectors with a TEV cutting site between the tag and desired protein domain. The plasmids were subsequently transformed into *Escherichia coli* BL21 (DE3) cells, which were grown at 37°C in LB medium supplemented with 50 mg/L Kanamycin until OD_600_ reached ~1.2–1.6. The proteins were overexpressed by the addition of IPTG to a final concentration of 0.5 mM. Post induction incubation overnight at 16°C, the culture was harvested by centrifugation at 4,000 rpm for 10 min. Cell pellets were re-suspended in lysis buffer (100 mM phosphate buffer pH 8.0, 500 mM NaCl, 10 mM β-ME, 10% glycerol and 25 mM imidazole pH 8.0). The suspension was lysed by sonication and centrifuged at 18,000 rpm at 4°C for 20 minutes. The supernatant was loaded onto a nickel-affinity column pre-equilibrated with wash buffer (50 mM Tris at pH 8.0 and 500 mM NaCl). The protein was washed with wash buffer with various concentrations of imidazole and finally eluted with the wash buffer in the range of 100–500 mM imidazole. The eluted protein was further purified by size-exclusion chromatography using the buffer (20 mM HEPES pH 7.1, 150 mM NaCl and 1 mM DTT). The protein concentrated to 10 mg/ml and stored under −80°C for further use.

#### Protein labeling

For the protein labeling reaction, 1 mg protein was used and the protein concentration was at least 2 mg/mL. An appropriate volume of 1 M sodium bicarbonate was added to the protein solution to achieve a final concentration of 100 mM. Then, 10 μL amine-reactive Atto 488 dye (Jena Bioscience) was added to the protein solution, and the solution was incubated at room temperature for one hour with shaking using a rotator tube revolver (Thermo Scientific). After incubation, gel filtration using Sephadex G-15 (Cytiva) was used to separate the conjugated protein and unreacted dye. The concentration and the Degree of Labeling (DOL) of conjugated protein fractions were calculated by using Equations 1 and 2. The DOL is the average number of fluorophore molecules per molecule of protein, and the ideal value for labeling reaction should be approximately 1 molecule of fluorophore per 200 amino acids based on the supplier’s protocol.

(Equation 1) $$\text{Conjugated}\;\text{protein}\;\text{concentration}\;(mg/mL)=\left(\left(A_{280}-A_{max}*CF\right)/\varepsilon_{280}\right)\;*MW_{\text{protein}}$$

(Equation 2) $$\text{Degree}\;\text{of}\;\text{Labeling}\;(DOL)=\left(A_{max}*\;\varepsilon_{280}\right)/\left(\left(A_{280}-A_{max}\;*CF\right)\;*\;\varepsilon_{max}\right)$$

$A_{280}$ = Absorbance of the conjugate solution measured at 280 nm.

$A_{\max}$ = Absorbance of the conjugate solution measured at $\lambda_{\mathrm{excitation}}$

$\lambda_{\text{excitation}}$ = Excitation maximum at wavelength 501 nm.

CF (Correction factor) = 0.1

$\varepsilon_{280}$ = Number of Tryptophan *5500 + Number of Tyrosine *1490 + Number of Cysteine *125.

${\mathrm{MW}}_{\mathrm{protein}}$ = Protein molecular weight

$\varepsilon_{\text{max}}$ (Extinction coefficient) = 90,000 mol^−1^ cm^−1^

#### Mycobacteria binding assay

For the *M. smegmatis* binding assay, 500 μL bacterial culture was centrifuged at 5000 rpm (Thermo Scientific, Fresco^™^ 21 Microcentrifuge) for 4 minutes to obtain the bacterial pellet. Then, 120 μL 1X PBS was used to resuspend the pellet before 40 μg of labeled protein was added to 120 μL bacteria. The sample was incubated at room temperature for 30 minutes shaking using a rotator tube revolver (Thermo Scientific). After incubation, the mixture was centrifuged in 5000 rpm for 4 minutes to obtain the pellet. The pellet was washed two times using 1X PBS with 0.5% Tween 20 and the fluorescent signal was observed using a confocal microscope (ZEISS LSM 900 with Airyscan 2 Inverted Confocal Microscope). For experiments with *E. coli*, 200 μL bacterial culture was used for the binding assay and all the steps are the same as with *M. smegmatis*.

#### LAM binding using glycan microarray

Glycan microarray slides^66^ were pre-hydrated with TMST buffer [25 mM Tris-HCl (pH 7.4), 0.15 mM NaCl, 2 mM CaCl2, 2 mM MgCl2, 0.05% Tween 20] for 5 min at room temperature, then blocked with TMST–BSA buffer [25 mM Tris-HCl (pH 7.4), 0.15 mM NaCl, 2 mM CaCl2, 2 mM MgCl2, 0.05% Tween 20, 1% BSA] at 37°C for 30 min. After blocking, protein samples (100 μL, 100 μg/mL in TMST–BSA buffer) were loaded into the wells of the slide. The wells were covered with aluminum adhesive seal and incubated for 2 h at room temperature with shaking at 150 rpm to allow for protein binding. Protein samples were pipetted out, and the slides were washed three times with TMST buffer. The primary antibody (100 μL of anti-6X his epitope tag mouse monoclonal IgG1 (kappa) antibody) was loaded into the wells. Wells were then sealed with adhesive tape then incubated for 45 min at room temperature with shaking at 150 rpm. The antibody was pipetted out, and the slides were washed three times with TMST buffer. The secondary antibody (100 μL of Cy3-conjugated rabbit anti-mouse IgG1 (Gamma 1 chain) antibody) was then loaded into the wells. The wells were then sealed with adhesive tape then incubated for 45 min at room temperature with shaking at 150 rpm. The antibody was pipetted out, and the slides were washed sequentially with TMST buffer, followed by TMS buffer [25 mM Tris-HCl (pH 7.4), 0.15 mM NaCl, 2 mM CaCl2, 2 mM MgCl2]. Prior to scanning, slides were dried by centrifugation.

#### Microarray scan and data analysis

Glycan microarrays were scanned at 10 μM pixel size using GenePix^®^ 4000B Molecular Devices Scanner. The fluorescent signal from th Cy3 dye was detected at 532 nm. Laser power was set at 100% and photomultiplier tube gain was set at 500. Pixel density (intensity) of each spot was quantified using GenePix^®^ Pro 7 Software. The fluorescence intensities for each spot and the its local background were calculated. Relative fluorescence units were computed by subtracting the local background signal from the signal of each spot. The mean of triplicate readings was calculated using Microsoft Excel.

#### Adsorption assay

The adsorption assay performed is based on a previously described method.^68^ In brief, *Mycobacterium smegmatis mc*^*2*^*155* strains were cultured in 7H9 medium with 10% ADC, 0.5% Tween 80, and 1 mM CaCl_2_ to an OD_600_ of 0.2, equivalent to a concentration of 6 × 10^6^ CFU/mL. A 10 mL of the bacterial culture was collected, centrifuged at 5000 × g for 5 minutes, and the supernatant was discarded. The bacterial pellet was resuspended in 1ml of fresh 7H9 medium with 10% ADC, and 1 mM CaCl_2_. The bacterial suspension was then mixed with mycobacteriophage Douge at a multiplicity of infection (MOI) of 0.001 in a 12-well polystyrene plate. The mixture was incubated at 37°C while shaking at 200 rpm. Samples of 50 μL were collected at 0, 10, 20, 30, 40, 50 and 60 minutes, centrifuged at 11,000 × g for 3 minutes, and the supernatant was used to quantify the number of free (unadsorbed) mycobacteriophages through plaque assays.

#### Double agar spot test

*Mycobacterium smegmatis* mc^2^155 or *ΔlamH* was grown in fresh 7H9 medium (10% ADC) to an OD_600_ of 2.0. A 1 mL aliquot of each bacterial culture was mixed with 10 mL of soft 7H9 agar (0.35% agar) and poured onto 7H10 agar plates to create a uniform bacterial lawn. Serial 10-fold dilutions of the Douge (5×10^9^ PFU/mL) were spotted in 3 μL drops on the surface of the bacterial lawn. The plates were incubated at 37°C for 24–48 hours until confluent bacterial growth was observed. To assess the effect of maltodextrin (DP10–40) on phage activity, 10 μL of phage stock was pre-mixed with 90 μL of varying concentrations of maltodextrin (Elictyl, France) in phage buffer, incubated at room temperature for 30 minutes, and subsequently diluted 10-fold in phage buffer.

#### Coarse-grained simulation

A particle-based 2D coarse-grained model of mycobacteriophage Douge was generated to explore how the tail bending affects for the baseplate-host interaction. We decomposed the molecular structure of Douge into three regions: capsid, tail, and baseplate based on the cryo-EM data, then converted it into a coarse-grained model. The capsid was represented by a hexagonal lattice with a lattice constant of 43.75 Å. The tail tube was described by a bead-spring model, with a bead representing a ring unit, and the elastic modulus of the phage is set to 0.6 GPa to simulate its mechanical properties. The baseplate is represented by two beads to mimic the trimeric structure of the baseplate domain in 2D (Figure S12A). The simulation system included the coarse-grained model of Douge, solution particles, and the cell surface. The simulations were performed using LAMMPS.^103^ The size of the simulation box measured 5,000 Å × 5,000 Å square and contained a total of 5,293 particles and the detailed simulation parameters are tabulated (see Table S5). A fixed boundary condition was applied at the cell surface. The initial configuration of the phage was set to a horizontally, with the head region within the potential cutoff range, to investigate whether the phage can successfully land and stand on the cell surface. We simulated two types of phage models (with flexible and rigid tails) to study their effects on the successful adhesion of the baseplate to the cell surface. The simulation consisted two steps. In the first step, the phage was fixed and solution particles are equilibrated. In the second step, the phage was released for 300 nanoseconds. The second step was repeated 100 times for both phage models. Here, we computed evolution of the distance between the baseplate and cell surface as a function of simulation time and quantify the successful adhesion rate for both phage models.

### QUANTIFICATION AND STATISTICAL ANALYSIS

Statistical analysis was performed on adsorption experiment data from three independent biological replicates. PFU counts from each time point were normalized to percentages, with the initial PFU at time zero set as 100%. Differences between wild-type and Δ*lamH M. smegmatis* strains at each time point were evaluated using two-way ANOVA with Šídák’s multiple comparisons test in GraphPad Prism version 10.4.1. A p-value <0.05 was considered statistically significant (**p < 0.01; ****p < 0.0001).

## Supplementary Material

Supplemental Info

Video S3

Video S2

Video S1

SUPPLEMENTAL INFORMATION

Supplemental information can be found online at https://doi.org/10.1016/j.celrep.2025.116057.

## Figures and Tables

**KEY RESOURCES TABLE:** 

REAGENT or RESOURCE	SOURCE	IDENTIFIER

Antibodies		

Rabbit Anti-Mouse IgG1 (Gamma 1 chain) Antibody Cy3 Conjugated	Rockland	Cat# 610-404-040
Anti-6X His Epitope Tag (Mouse) Monoclonal Antibody	Rockland	Cat# 200-301-382
Bacterial and virus strains		
*Mycobacterium smegmatis* mc^2^155	Snapper et al. (1990)^84^	GenBank: NC_008596
*Mycobacterium smegmatis* mc^2^155 ΔlamH	Franklin et al. (2024)^68^	N/A
*Escherichia coli* DE3 BL21	YB Biotech	Cat# FYE207-40VL
*Escherichia coli* DH5α Competent Cells	YB Biotech	Cat# FYE678-80
Mycobacteriophage Douge	This study (Hsinchu, Taiwan)	GenBank: PQ184818.1; https://www.ncbi.nlm.nih.gov/nuccore/PQ184818.1

Chemicals, peptides, and recombinant proteins		

CaCl_2_	Sigma-Aldrich	Cas# 10043
Cycloheximide	Sigma-Aldrich	Cas# 66-81-9
Carbenicillin	Sigma-Aldrich	Cas# 4800-94-6
Difco Middlebrook 7H9 broth	BD	SKU 271310
Glycerol	Cyrusbioscience	Product# 101-56-81-5
Agar	Cyrusbioscience	Product# 9002-18-0
middlebrook 7H10 Agar Base	Himedia	SKU M199
NaCl	Cyrusbioscience	Product# 101-7647-14-5
poly(ethylene glycol) average mw 8000	Sigma-Aldrich	Cas# 25322-68-3
Tris-HCl	Cyrusbioscience	Product# 1185-53-1
Magnesium Chloride Solution	Sigma-Aldrich	Cas# 7786-30-3
Cesium Chloride	ThermoFischer Scientific	Cas# 7647-17-8
D-glucose anhydrous	Fisher Chemicals	Cas# 50-99-7
Bovine Serum Albumin	Sigma-Aldrich	Cas# 9048-46-8
Chloroform	Ducsan	UN1888
Phenol, Molecular Biology Grade	Merck	Cas# 108-95-2
Ethanol, 99.8%	Fisher Chemicals	Cas# 64-17-5
Tween 80	Sigma-Aldrich	Product# P1754-500ML, Cas# 9005-65-6
Tween 20 (Polyoxyethylene 20 Sorbitan Monolaurate	J.T. Baker	Product# X251-07, Cas# 9005-64-5
Bovine Serum Albumin Fraction V	Roche	Product# 10735094001

Critical commercial assays		

Zeba Spin Desalting Columns	ThermoFischer Scientific	Product# 89890
Atto 488 Protein Labeling Kit	Jena Bioscience	Cat# FP-201-488
Cy5 Protein Labeling Kit	Jena Bioscience	Cat# FP-201-CY5
Alexa Fluor^™^ 532 Protein Labeling Kit	ThermoFischer Scientific	Cat# A10236

Deposited data		

Cryo-EM structure of Mycobacteriophage Douge genome–packed capsid (gp8 and gp113)	This Study	EMDB: EMD-39973
Focused refinement cryo-EM map of Mycobacteriophage Douge genome–packed capsid (Penton region: gp8)	This Study	EMDB: EMD-39974
Focused refinement cryo-EM map of Mycobacteriophage Douge genome–packed capsid (P-hexon region: gp8 and gp113)	This Study	EMDB: EMD-39975
Focused refinement cryo-EM map of Mycobacteriophage Douge genome–packed capsid (C-hexon region: gp8 and gp113)	This Study	EMDB: EMD-39976
Composite cryo-EM map of Mycobacteriophage Douge genome–packed capsid (Penton, P-hexon and C-hexon region: gp8 and gp113)	This Study	EMDB: EMD-39977
Cryo-EM structure of Mycobacteriophage Douge genome–free capsid (gp8)	This Study	EMDB: EMD-39978
Focused refinement cryo-EM map of Mycobacteriophage Douge genome–free capsid (Penton region: gp8)	This Study	EMDB: EMD-39979
Focused refinement cryo-EM map of Mycobacteriophage Douge genome–free capsid (P-hexon region: gp8)	This Study	EMDB: EMD-39980
Focused refinement cryo-EM map of Mycobacteriophage Douge genome–free capsid (C-hexon region: gp8)	This Study	EMDB: EMD-39981
Composite cryo-EM map of Mycobacteriophage Douge genome–free capsid (Penton, P-hexon and C-hexon region: gp8)	This Study	EMDB: EMD-39982
Cryo-EM structure of Mycobacteriophage Douge genome–packed connector-vertex (gp5, gp8, gp9, gp10, gp12, gp13 and gp113)	This Study	EMDB: EMD-60714
Cryo-EM structure of Mycobacteriophage Douge genome–packed connector (gp5, gp9, gp10, gp12 and gp13)	This Study	EMDB: EMD-39983
Cryo-EM structure of Mycobacteriophage Douge genome–packed vertex (gp8 and gp113)	This Study	EMDB: EMD-39984
Focused refinement cryo-EM map of Mycobacteriophage Douge genome–packed connector (gp5 and gp9)	This Study	EMDB: EMD-39985
Focused refinement cryo-EM map of Mycobacteriophage Douge genome–packed connector (gp10, gp12 and gp13)	This Study	EMDB: EMD-39986
Focused refinement cryo-EM map of Mycobacteriophage Douge genome–packed connector (gp8 and gp113)	This Study	EMDB: EMD-39987
Composite cryo-EM map of Mycobacteriophage Douge genome–packed connector-vertex (gp5, gp9, gp10, gp12, gp13, gp8 and gp113)	This Study	EMDB: EMD-39988
Cryo-EM structure of Mycobacteriophage Douge genome–free connector vertex (gp5, gp8, gp9, gp10, gp12 and gp13)	This Study	EMDB: EMD-60715
Cryo-EM structure of Mycobacteriophage Douge genome–free connector (gp5, gp9, gp10, gp12 and gp13)	This Study	EMDB: EMD-39989
Cryo-EM structure of Mycobacteriophage Douge genome–free vertex (gp8)	This Study	EMDB: EMD-39990
Focused refinement cryo-EM map of Mycobacteriophage Douge genome–free connector (gp5 and gp9)	This Study	EMDB: EMD-39991
Focused refinement cryo-EM map of Mycobacteriophage Douge genome–free connector (gp10, gp12 and gp13)	This Study	EMDB: EMD-39992
Focused refinement cryo-EM map of Mycobacteriophage Douge genome–free connector (gp8)	This Study	EMDB: EMD-39993
Composite cryo-EM map of Mycobacteriophage Douge genome–free connector-vertex (gp5, gp9, gp10, gp12, gp13 and gp8)	This Study	EMDB: EMD-39994
Cryo-EM structure of Mycobacteriophage Douge genome–packed tail tube (gp13)	This Study	EMDB: EMD-60024
Cryo-EM structure of Mycobacteriophage Douge genome–free tail tube (gp13)	This Study	EMDB: EMD-39995
Cryo-EM structure of Mycobacteriophage Douge baseplate (gp13, gp17, gp23, gp16, gp18 and gp20)	This Study	EMDB: EMD-39996
Focused refinement cryo-EM map of Mycobacteriophage Douge baseplate (gp13)	This Study	EMDB: EMD-39997
Focused refinement cryo-EM map of Mycobacteriophage Douge baseplate (gp17 and gp23)	This Study	EMDB: EMD-39998
Focused refinement cryo-EM map of Mycobacteriophage Douge baseplate (gp16, gp18 and gp20)	This Study	EMDB: EMD-39999
Composite cryo-EM map of Mycobacteriophage Douge baseplate (gp13, gp17, gp23, gp16, gp18 and gp20)	This Study	EMDB: EMD-60000
Cryo-EM structure of Mycobacteriophage Douge Central fiber (gp20)	This Study	EMDB: EMD-60001
Focused refinement cryo-EM map of Mycobacteriophage Douge Central fiber (gp20, top region)	This Study	EMDB: EMD-60002
Focused refinement cryo-EM map of Mycobacteriophage Douge central fiber (gp20, bottom region)	This Study	EMDB: EMD-60003
Composite cryo-EM map of Mycobacteriophage Douge central fiber (gp20)	This Study	EMDB: EMD-60004
Cryo-EM structure of Mycobacteriophage Douge complete baseplate (gp13, gp17, gp23, gp16, gp18 and gp20)	This Study	EMDB: EMD-60005
Cryo-EM structure of Mycobacteriophage Douge genome–packed capsid (gp8 and gp113)	This Study	PDB: 8ZDH
Cryo-EM structure of Mycobacteriophage Douge genome–released capsid (gp8)	This Study	PDB: 8ZDI
Cryo-EM structure of Mycobacteriophage Douge genome–packed connector (gp5, gp9, gp10, gp12 and gp13)	This Study	PDB: 8ZDJ
Cryo-EM structure of Mycobacteriophage Douge genome–packed vertex (gp8 and gp113)	This Study	PDB: 8ZDK
Cryo-EM structure of Mycobacteriophage Douge genome–released connector (gp5, gp9, gp10, gp12 and gp13)	This Study	PDB: 8ZDL
Cryo-EM structure of Mycobacteriophage Douge genome–released vertex (gp8)	This Study	PDB: 8ZDM
Cryo-EM structure of Mycobacteriophage Douge genome–packed tail tube (gp13)	This Study	PDB: 8ZEA
Cryo-EM structure of Mycobacteriophage Douge genome–free tail tube (gp13)	This Study	PDB: 8ZDN
Cryo-EM structure of Mycobacteriophage Douge baseplate (gp13, gp17, gp23, gp16, gp18 and gp20)	This Study	PDB: 8ZDO
Cryo-EM structure of Mycobacteriophage Douge Central fiber (gp20)	This Study	PDB: 8ZDP
Cryo-EM structure of Mycobacteriophage Douge complete baseplate (gp13, gp17, gp23, gp16, gp18 and gp20)	This Study	PDB: 8ZDQ

Recombinant DNA		

pET15-His-GST-Douge_8-Ig-like (MCP-Ig-like domain)	This Study	N/A
pET9-His-MBP-Douge_13-Ig-like (TTP-Ig-like domain)	This Study	N/A
pET9-His-GFP-Douge_gp20_D1 (CFP-NTD)	This Study	N/A
pET15-His-GFP-Douge_20-3 (CFP-tip-CTD)	This Study	N/A
pET9-His-Gp23-D1 (BppU-NRD)	This Study	N/A
pET9-His-GFP-Douge_Gp23_D2 (BppU - CBM)	This Study	N/A

Software and algorithms		

GS *De Novo* Assembler (Newbler) v2.9	Roche/454 Life Sciences, Branford, CT	N/A
DNA Master v 5.23.6	J.G. Lawrence, University of Pittsburgh	http://cobamide2.bio.pitt.edu
BLASTp	Altschul et al.^85^	https://blast.ncbi.nlm.nih.gov/Blast.cgi
HHpred	Söding et al.^86^	https://toolkit.tuebingen.mpg.de/tools/hhpred
PECCAN	NA	http://pecaan.kbrinsgd.org
ARAGORN v1.1 / v1.2.38	Laslett and Canback^87^	N/A
tRNAscan-SE v2.0	Chan and Lowe^88^	https://lowelab.ucsc.edu/tRNAscan-SE/
PhagesDB	Russell and Hatfull^43^	https://phagesdb.org
Phamerator	Cresawn et al.^89^	https://phamerator.org
MotionCor2	Zheng et al.^90^	https://emcore.ucsf.edu/ucsf-software
CTFFIND4.1	Rohou and Grigorieff^91^	https://grigoriefflab.umassmed.edu/ctffind4
CryoSPARC	Punjani et al.^92^	https://cryosparc.com/
Alphafold2	Jumper et al.^44^	https://github.com/google-deepmind/alphafold
Chimera	Pettersen et al.^93^	https://www.cgl.ucsf.edu/chimera/
ChimeraX	Meng et al.^94^	https://www.cgl.ucsf.edu/chimerax/
Coot	Emsley and Cowtan^95^	https://www2.mrc-lmb.cam.ac.uk/personal/pemsley/coot/
Phenix	Afonine et al.^96^	https://phenix-online.org/
DeepEMhancer	Sanchez-Garcia et al.^97^	https://github.com/rsanchezgarc/deepEMhancer
DeepMainmast	Terashi et al.^98^	https://kiharalab.org/emsuites/deepmainmast.php
IMOD v4.9.12	Mastronarde and Held^99^	https://bio3d.colorado.edu/imod/
Topaz v0.2.5	Bepler et al.^100^	https://github.com/tbepler/topaz
tom_deconv	Tegunov and Cramer^101^	https://github.com/dtegunov/tom_deconv
Amira software 2022.1	Martinez-Sanchez et al.^102^	Thermo Fisher Scientific, Hillsboro, OR, USA
LAMMPS	Thompson et al.^103^	https://www.lammps.org/#gsc.tab=0
GraphPad Prism version 10.4.1	GraphPad	https://www.graphpad.com/

## Highlights

- Atomic-resolution structure of the complete mycobacteriophage Douge is determined
- An asymmetric interface between the capsid-vertex and portal-adaptor subcomplex is observed
- Glycan-binding preferences of Douge receptor-binding proteins are identified
- Cryo-ET snapshots capture distinct stages of Douge infecting *Mycobacterium smegmatis*
