# Supplementary material for: Cryo-EM and cryo-ET reveal the molecular architecture and host interactions of mycobacteriophage Douge
Source: Cell Rep. Author manuscript; Available in PMC 2026 Mar 29. (PMC13033339; doi:10.1016/j.celrep.2025.116057)
Supplement: Supplemental Info [file NIHMS2152381-supplement-Supplemental_Info.pdf]

Cell Reports, Volume *44*

Supplemental information

Cryo-EM and cryo-ET reveal the molecular architecture and host interactions of mycobacteriophage Douge

Jitendra Maharana, Chun-Hsiung Wang, Li-An Tsai, Yi-Ting Liao, Cheng-Han Yang, Melvin C. Shen, Lourriel S. Macale, Thang Ngoc Tran, Joemark Narsico, Ronelito J. Perez, Sunil Kumar Tewary, Jian-Li Wu, Hong-You Lin, Shu-Wei Chang, Aaron Franklin, Patrick J. Moynihan, Deborah Jacobs-Sera, Krista G. Freeman, Graham F. Hatfull, Todd L. Lowary, and Meng-Chiao Ho

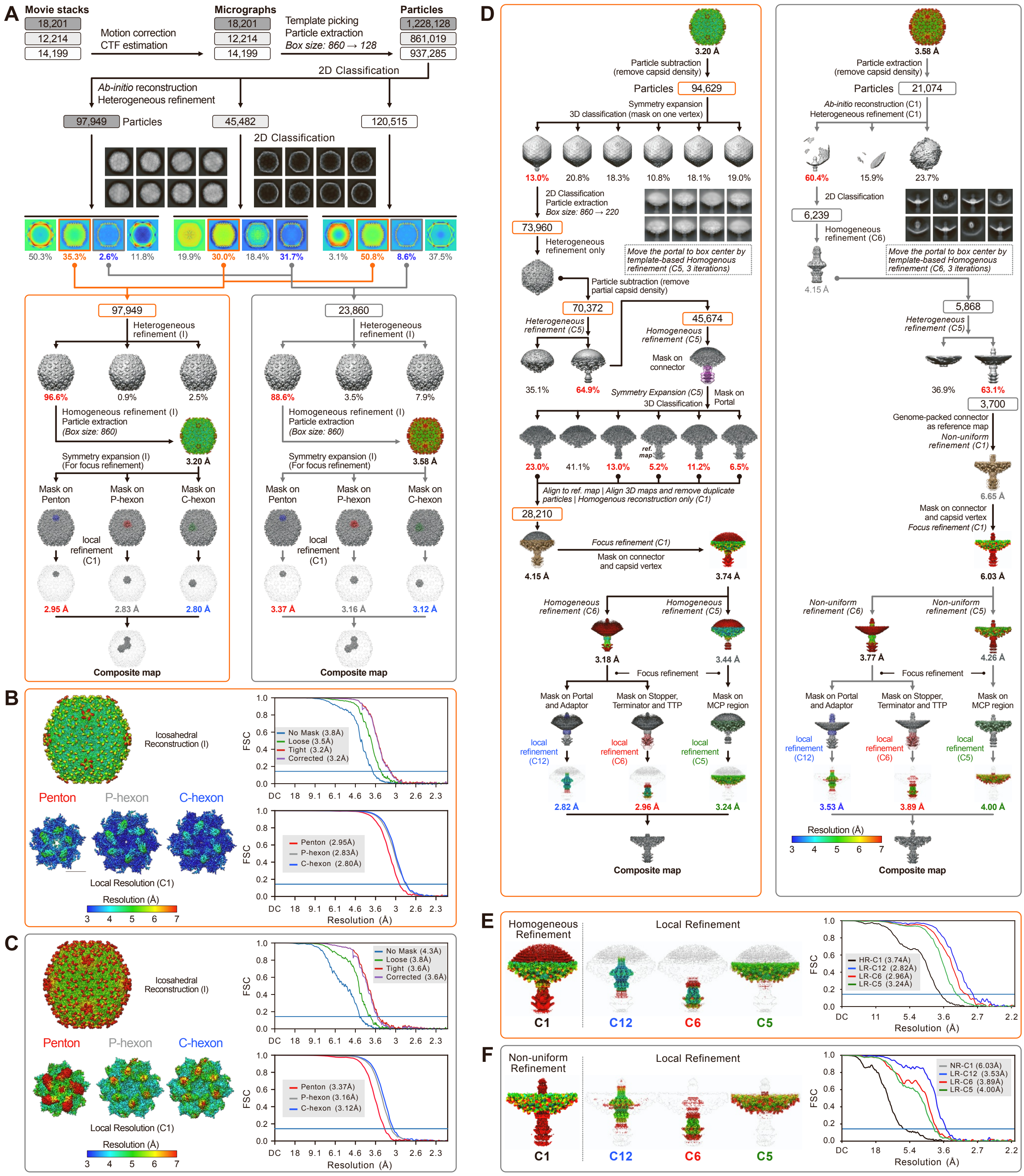

Figure S1. Cryo-EM Structure Determination Flowchart of the Douge Capsid and Connector regions.
(A) Cryo-EM data processing workflow for genome-packed and genome-free Douge capsids.
(B, C) Gold-standard FSC curves (FSC = 0.143) for the genome-packed (B) and genome-free (C) Douge capsids, showing icosahedral (I) refinement and local C1 symmetry refinement of the Penton, P-hexon, and C-hexon regions. Focus refinement masks are color-coded: blue (Penton, mask 1), red (P-hexon, mask 2), and green (C-hexon, mask 3).
(D) Cryo-EM data processing workflow for genome–packed and genome–free Douge connectors, with cryo-EM maps color-coded according to local resolutions.
(E, F) Gold-standard FSC curves (FSC = 0.143) for the genome–packed (E) and genome-free (F) connector regions. The genome–packed connector was refined using homogeneous C1 refinement, while the genome-free connector underwent non-uniform (C1) and local refinements with C12, C6, and C5 symmetries. Focus refinement masks are color-coded: blue for the portal and adaptor (mask 1), red for the stopper, terminator, and TTP (mask 2), and green for the MCP (mask 3).

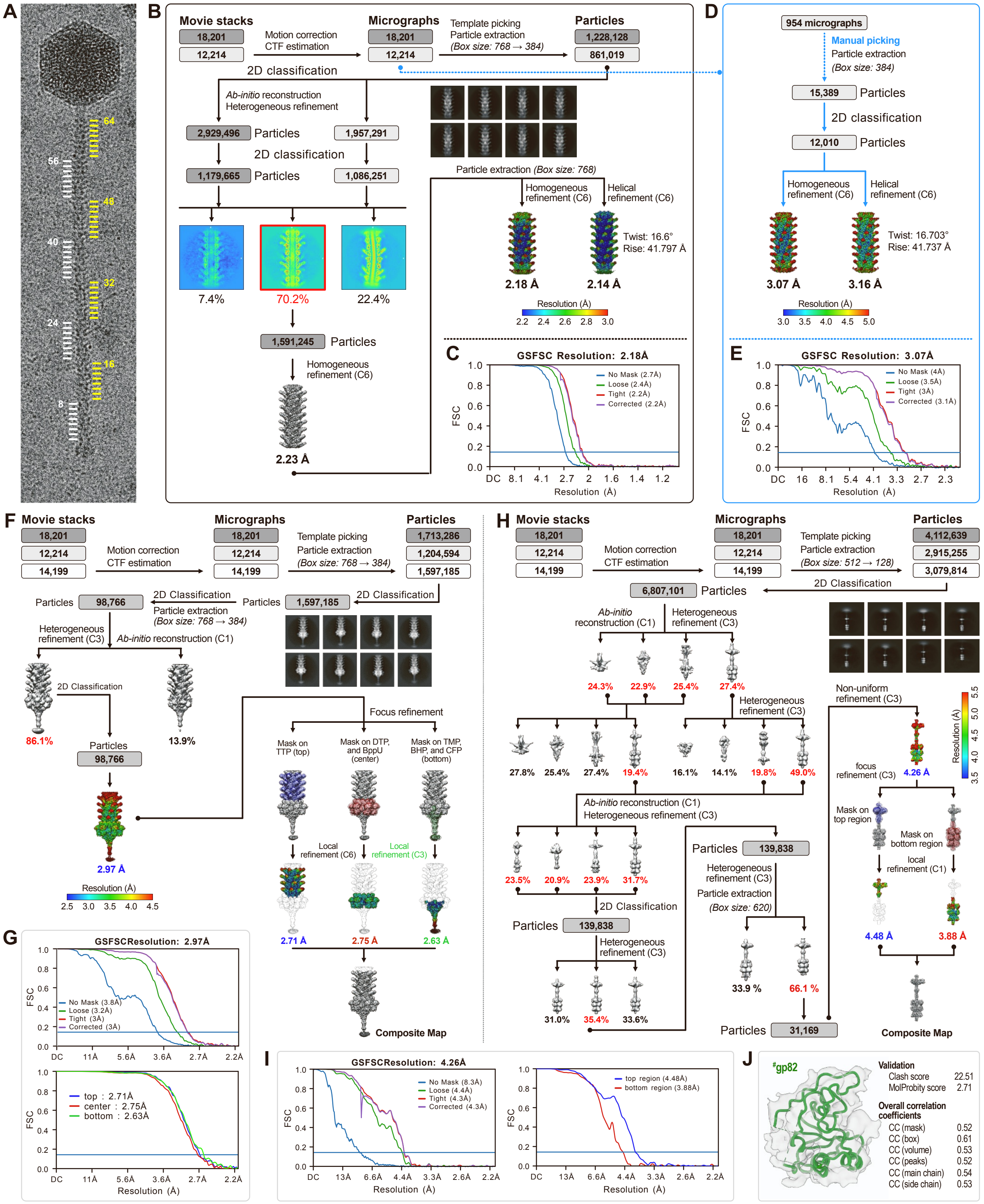

Figure S2. Cryo-EM Structure Determination Flowchart of Douge Tail tube, Baseplate and Central fiber.

(A) Cryo-electron micrograph of Douge, revealing 64 layers of TTP rings.

(B–E) Cryo-EM data processing workflows (B, D) and gold-standard FSC curves (C, E; FSC = 0.143) for genome-packed and genome-free tail tube structures, respectively.

(F) Cryo-EM data processing flowchart for the Douge baseplate.

(G) Comparative gold-standard FSC curves (FSC = 0.143) of baseplate maps obtained through C3 symmetry refinement and local refinement

(H) Cryo-EM workflow for the tail tip (or central fiber) region.

(I) Comparative gold-standard FSC curves (FSC = 0.143) of non-uniform and locally-refined maps for the central fiber regions.

(J) Overview of the hypothetical protein (#gp82) model within the map, with model validation reports.

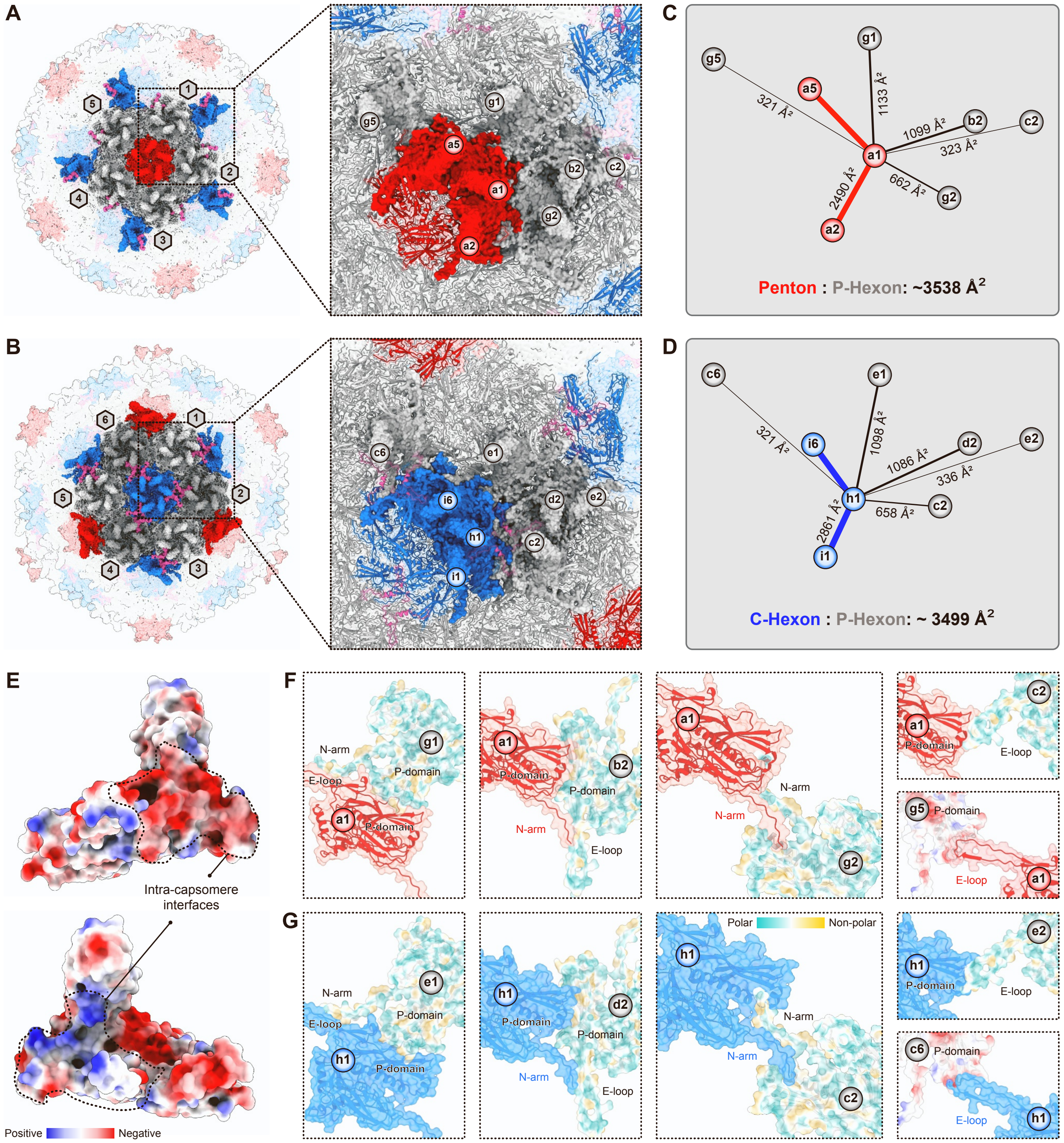

Figure S3. Overview of intra- and inter-capsomere Interaction Details.

(A, B) Overview of Douge capsid at the fivefold (A) and three-fold (B) axis, highlighting the association of one penton or C-hexon MCP subunit with adjacent subunits.

(C, D) Buried surface area for the penton-hexon (C) and hexon-hexon (D) interfaces.

(E) Electrostatic surface representation of one MCP subunit showing the negative (upper panel) and positive (lower panel) surface areas, which are involved in intra-capsomere interactions.

(F, G) Detailed views of the interactions between the penton (F) and C-hexon (G) MCP subunits with adjacent subunits. The penton and C-hexon MCP subunits are shown as red and blue cartoon-surface models, respectively, while surrounding MCP subunits are depicted with electrostatic or hydrophobic surface models.

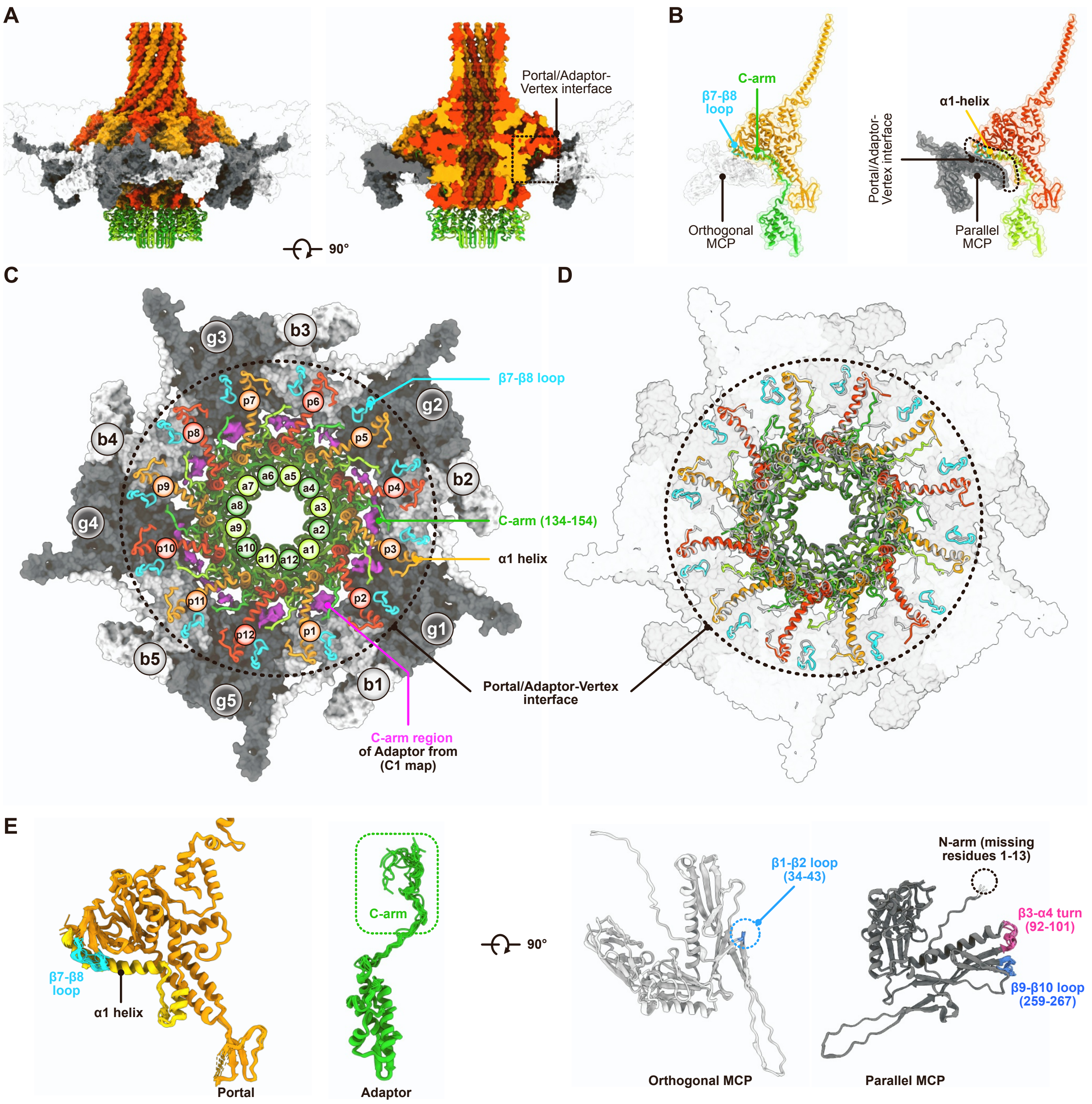

Figure S4. Portal–Vertex Symmetry Mismatched Interaction Interfaces.

(A) Overview of the C12 portal-adaptor fivefold vertex structure, with the Z-clipped map highlighting the symmetry-mismatched interface.

(B) Illustration of the 'orthogonal' and 'parallel' positioned MCP subunits assembling with portal and adaptor subunits.

(C) Overview of the capsid-vertex and portal-adaptor symmetry mismatched interfaces; the capsid-vertex is shown in white (orthogonal MCP) and dark gray (parallel MCP). The interacting regions of the portal (α1-helix) are shown in orange-red and orange, and the β7-β8 loop regions in cyan ribbon, while adaptor subunits are displayed as light and dark green cartoons. The traces of the C-arm of the adaptor from the C1 connector-vertex map is shown in magenta.

(D) Superimposed view of the dodecameric portal-adaptor structure (C1 model in color and C12 model in gray ribbon) highlighting dynamic regions. The portal/adaptor-capsid interface is marked with a black dotted circle.

(E) Conformational ensembles of the portal, adaptor, orthogonal, and parallel MCP subunits highlights the dynamic/missing regions.

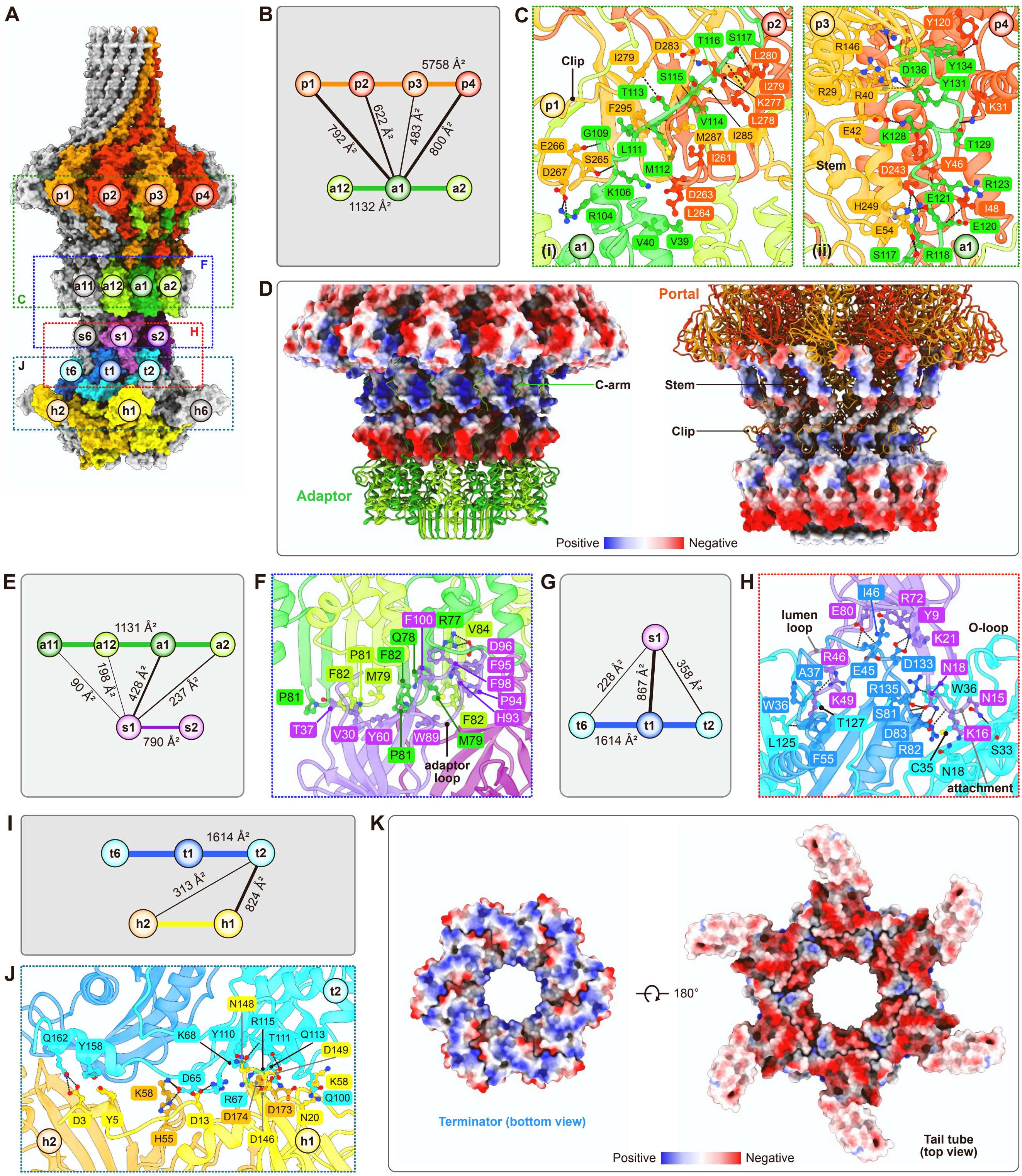

Figure S5. Connector Structure – Detailed Interaction Analysis.

(A) Surface diagram of the connector assembly showing interactions at the portal-adaptor, adaptor-stopper, stopper-terminator, and terminator-tail tube interfaces. Protein subunits are color-coded as follows: portal (p1–4) in orange/dark orange, adaptors (a1–a2 and a11–a12) in green/light green, stoppers (s1–s2 and s6) in purple/violet, terminators (t1–t2 and t6) in blue/cyan, and tail tube (h1–h2 and h6) in gold/light orange.

(B) Buried surface area between the portal and adaptor, with (C) a detailed view of their interactions.

(D) Electrostatic surface view of the portal and adaptor, highlighting the key interaction regions.

(E) Buried surface area between the adaptor and stopper, with (F) a detailed view of their interactions.

(G) Buried surface area between the stopper and terminator, with (H) a detailed view of their interactions.

(I) Buried surface area between the stopper and tail tube subunits, with (J) a detailed view of their interactions.

(K) Electrostatic surface potentials of the terminator (bottom view) and tail tube (top view), providing further insight into their interaction interfaces.

Key interacting residues are shown as ball-and-stick models, and intermolecular H-bonds are represented by black dotted lines.

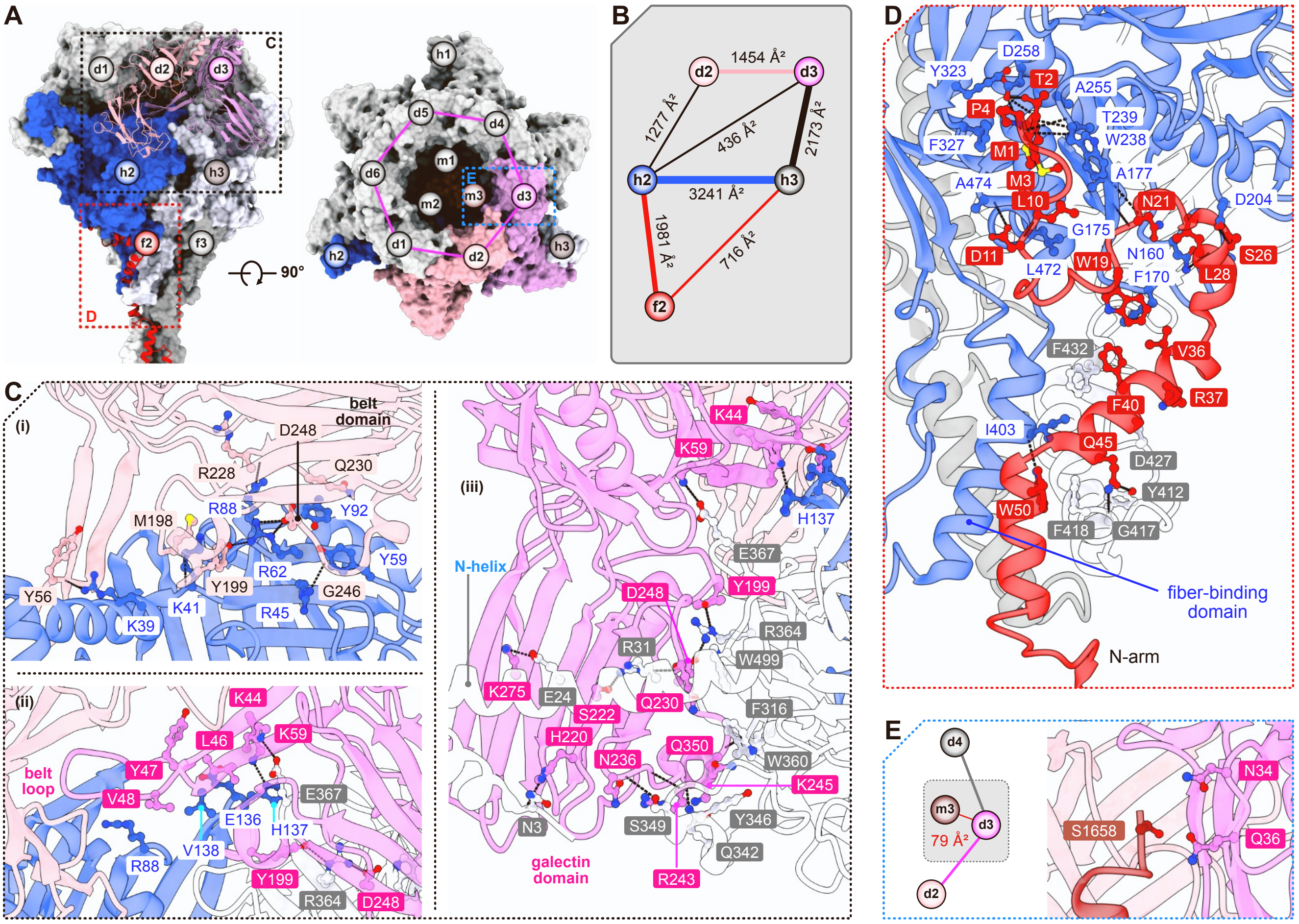

Figure S6. Overview of Symmetry-mismatched Interactions at the Baseplate.

(A) Top and side views of the C6-C3 symmetry mismatch assembly of the baseplate core, highlighting the structural arrangement of distal tail DTP (pink/magenta), BHP (blue/light gray), TMP (brown), and CFP (red ribbon) subunits.

(B) Illustration of the buried surface areas in the DTP-BHP and BHP-CFP associations.

(C) Close-up of the detailed molecular interactions between the DTP and BHP subunits, showing the specific residues involved in their binding interface and the types of contacts that stabilize the interaction.

(D) Detailed molecular interaction between the BHP and CFP subunits, displaying the interface residues and the network of hydrogen bonds and hydrophobic contacts that stabilize this symmetry mismatch interaction.

(E) Buried surface area and specific interactions between the TMP and DTP subunits, illustrating the interface residues that contribute to the stability of this assembly.

Key interacting residues are shown as ball-and-stick models, and intermolecular H-bonds are represented by black dotted lines.

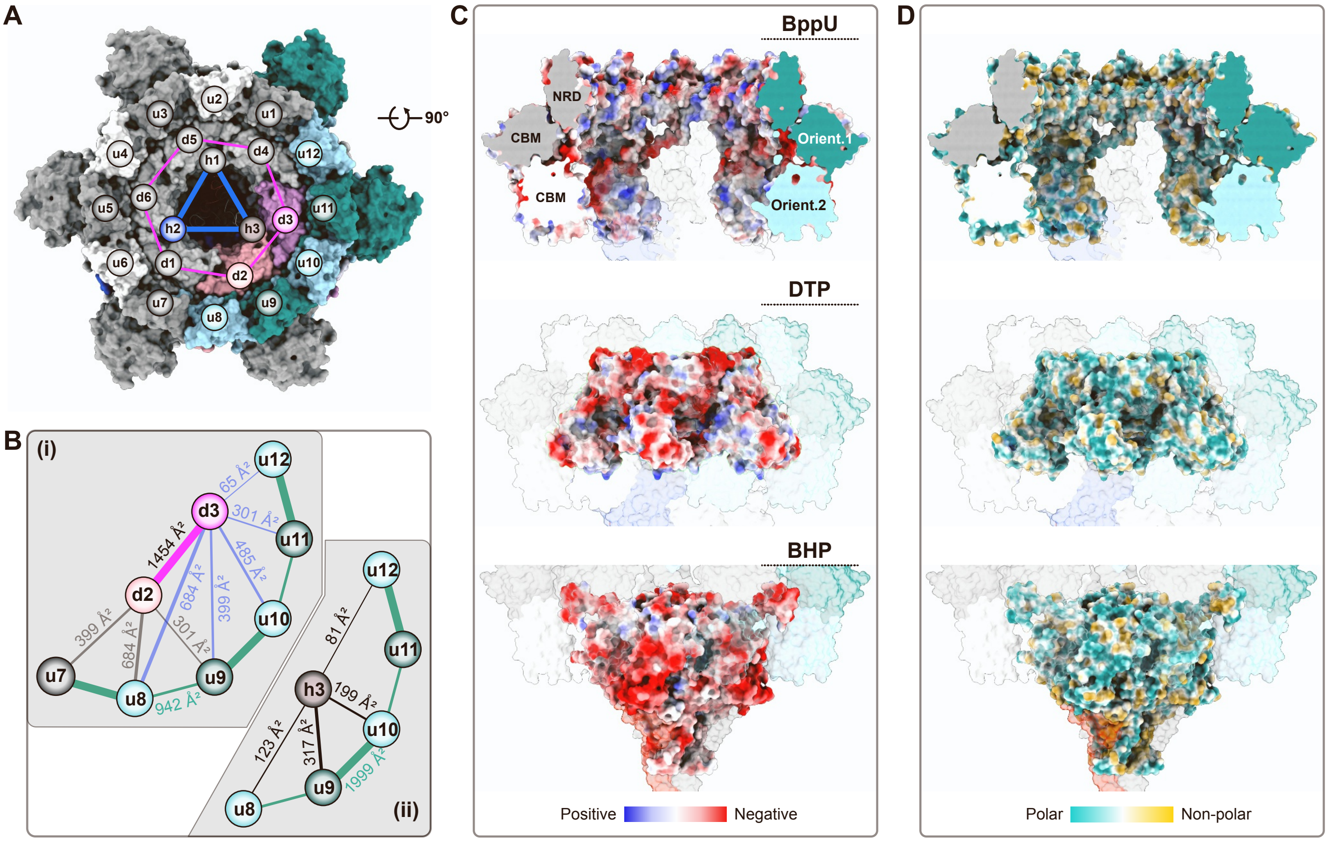

Figure S7. Structural Assembly of the DTP-BHP-BppU Complex.

(A) Top view of the BppU-DTP-BHP complex, illustrating the symmetrical arrangement of these components within the Douge baseplate.

(B) Spatial arrangements of the DTP-BppU and BHP-BppU interactions, with buried surface areas annotated to highlight the extent of contact regions between these components.

(C) Electrostatic and (D) lipophilicity surface distribution of the BppU (top), DTP (center), and BHP (bottom) subunits, with the cutaway views of BppU showing the arrangement of carbohydrate-binding modules (CBMs).

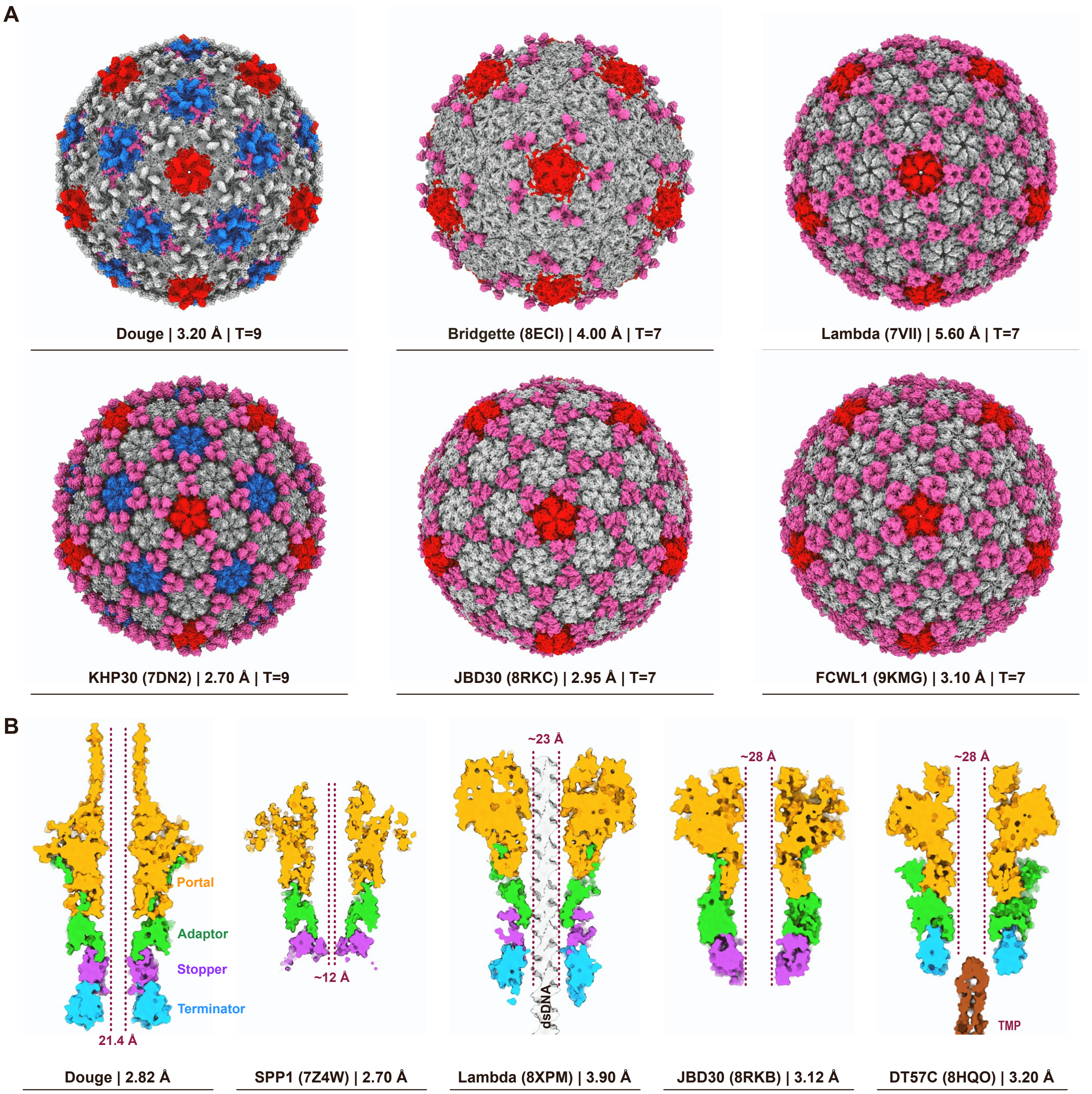

Figure S8. Structural Comparison of Siphophage Capsids and Connectors.
(A) Surface view of siphophage capsids, highlighting the arrangement of CCPs on MCP capsomeres in a 'T' organization. The penton, P-hexon, and C-hexon capsomeres are colored red, gray, and blue, respectively, while CCPs are shown in pink.
(B) Longitudinal section of siphophage connectors, illustrating their structural diversity. The portal, adaptor, stopper, terminator, and tape measure proteins are colored orange, green, violet, sky blue, and brown, respectively. The dsDNA in Lambda phage is displayed in gray. The narrowest diameter of the connector is marked with brown dotted lines and annotated.

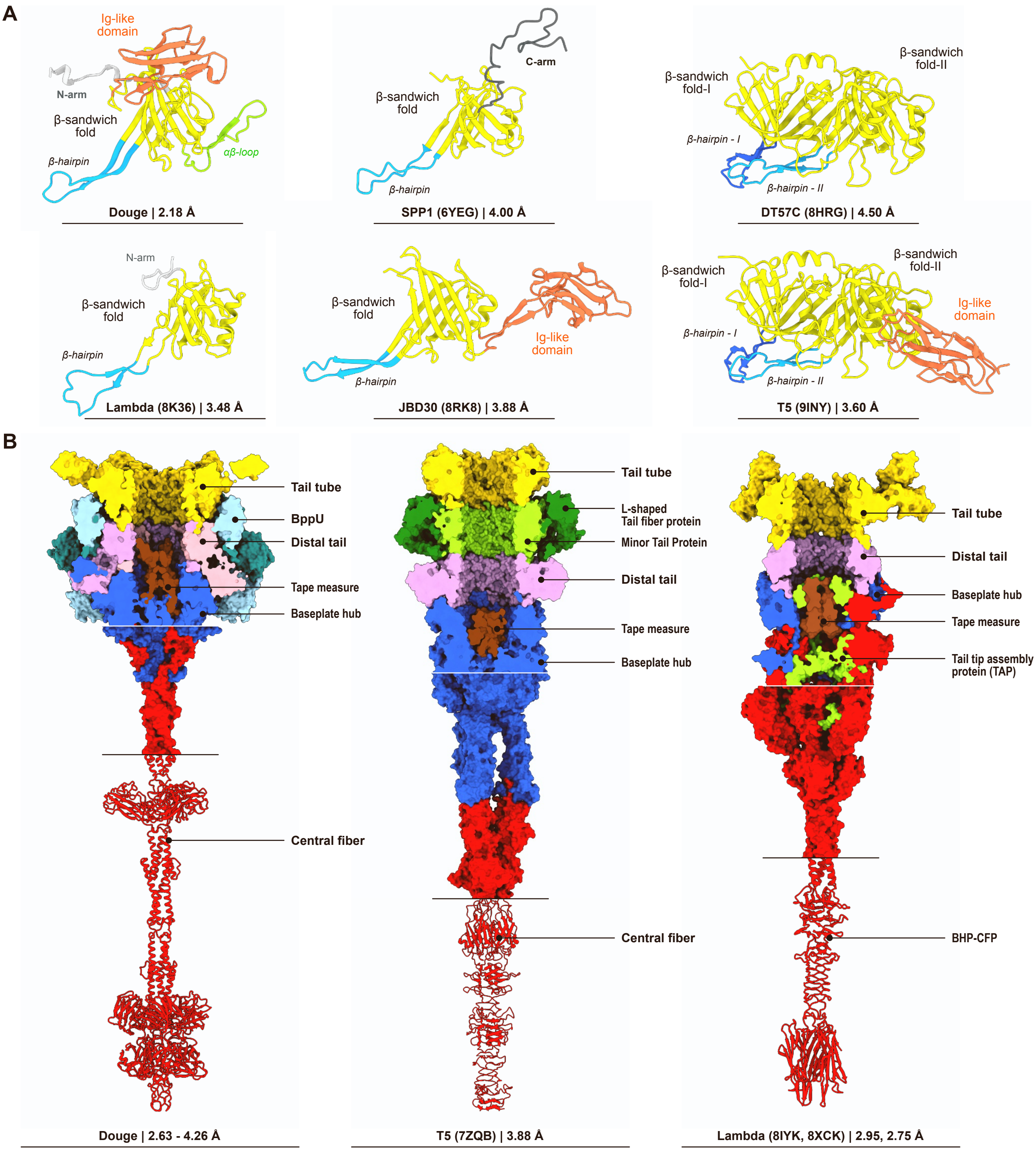

Figure S9. Structural Comparison of Siphophage Tail Tube and Baseplates.

(A) Comparative overview of siphophage TTP subunits, with annotated domains and regions. The β-sandwich fold and Ig-like domains are shown in yellow and brown, respectively, with other regions colored accordingly.

(B) Structural comparison of siphophage baseplates, highlighting their diverse arrangements. Different baseplate-associated proteins are distinctly colored and annotated.

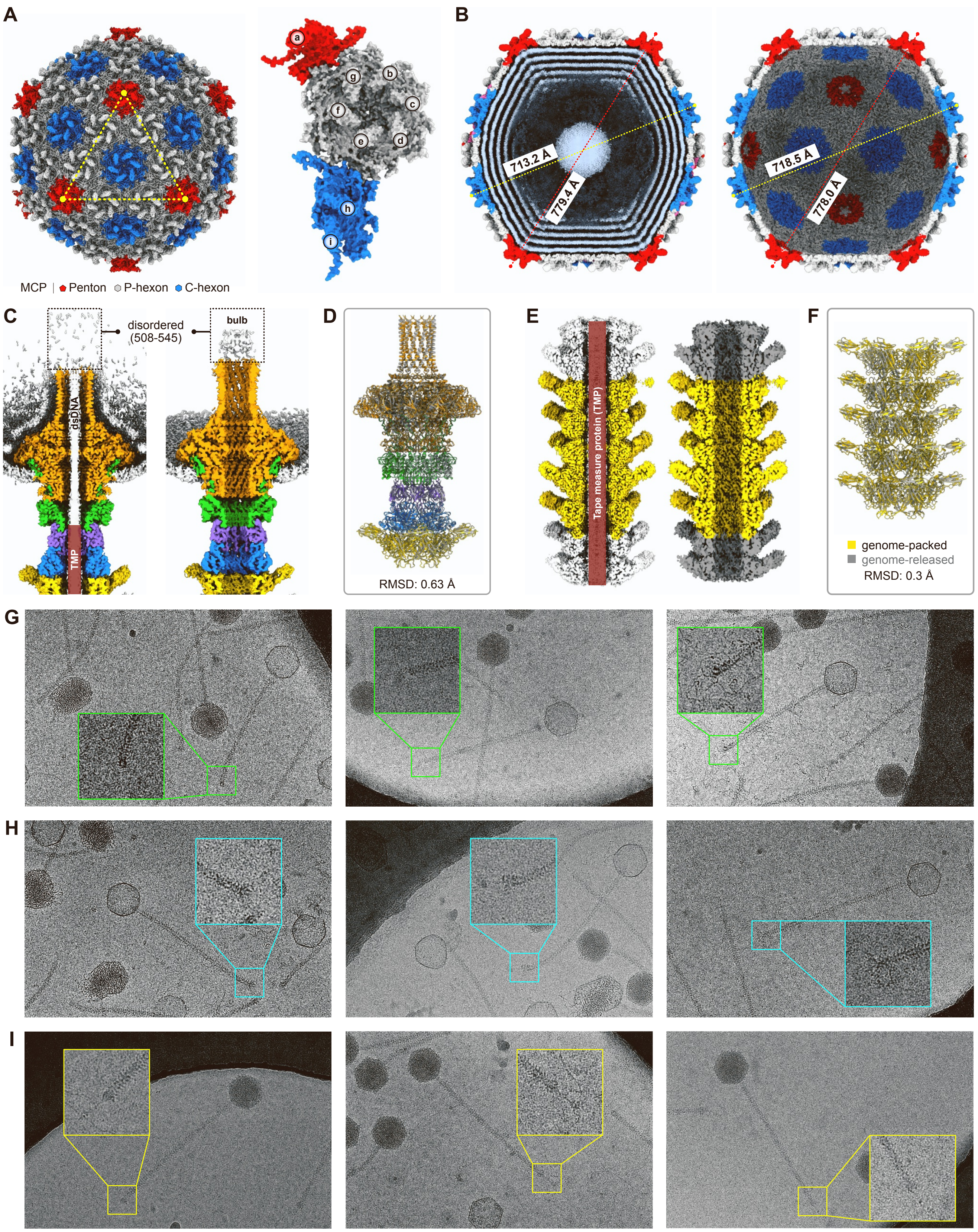

Figure S10. Structural Comparison of Genome-packed and Genome-free Douge Components.

(A) The genome-free Douge capsid in left, showing one asymmetric subunit in right panel.

(B) Z-clipped maps of genome-packed and genome-free capsids, highlighting dimers at the 3-fold and 5-fold axes.

(C) Z-clipped maps of the genome-packed connector, highlighting dsDNA and TMP, while the genome-free map shows bulb density with no density inside the connector channel.

(D) Superimposed view of the genome-packed (in color ribbon) and genome-free (in gray) connector structures, with an RMSD value of 0.63 Å.

(E) Comparative overview of cutaway maps of genome-packed and genome-free tail tubes. In the genome-packed tail tube channel, clear density corresponding to TMP is observed, whereas the genome-free tail tube lacks this density.

(F) Superimposed view of five stacked tail tube rings from the genome-packed (gold ribbon) and genome-free (gray) states, revealing no significant structural differences, with an RMSD of 0.3 Å.
(G-I) Representative cryoelectron micrographs showing three types of mycobacteriophages based on baseplate morphology: diamond-shaped baseplate with a long tip (genome-packed), crown-shaped (genome-free), and absent (genome-free). Solid capsids indicate genome-packed virions, while hollow capsids reflect a genome-free state; (G) Most mycobacteriophages lack visible baseplates in the genome-free state, (H) Fewer than 50 phages retain a crown-shaped baseplate despite being genome-free, (I) Genome-packed phages exhibit a needle-shaped baseplate.

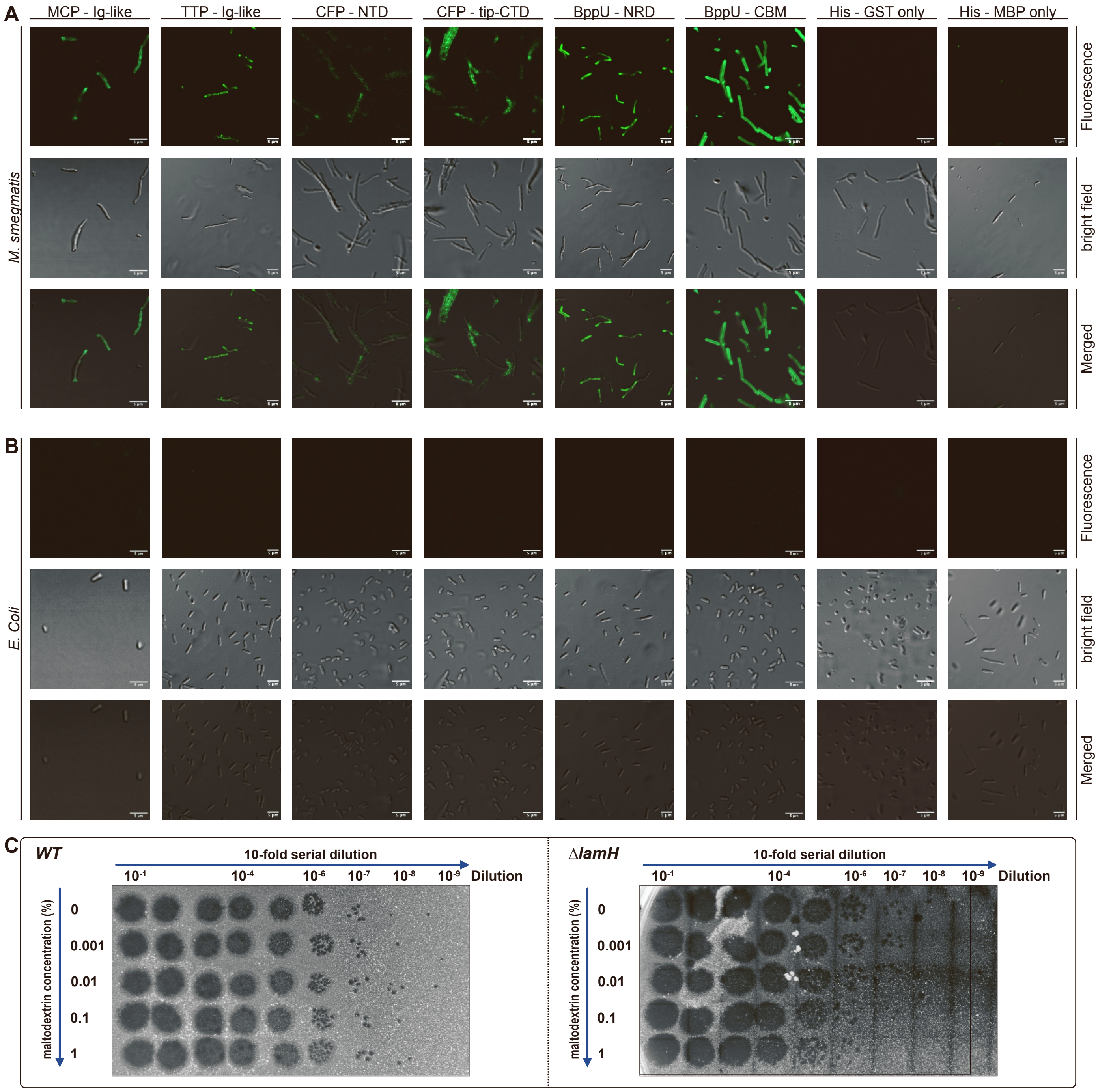

Figure S11. Cell attachment of Douge RBPs and phage infection in the presence of maltodextrin
(A, B) Confocal microscopy images of (A) *M. smegmatis* and (B) *E. coli* cell surface interaction with various Douge RBP modules labeled by green fluorescent dyes. MCP–Ig-like and BppU–CBM were tagged with His-GST, TTP-Ig-like with His-MBP, and CFP-NTD, CFP-tip-CTD, and BppU-NTD with His-tag; His-GST and His-MBP alone served as controls. Top panels show fluorescence, middle panels bright-field, and bottom panels merged images. Scale bar: 5 μm.
(C) Mycobacteriophage Douge forms plaques on *M. smegmatis* WT and Δ*lamH* mutant strains in the presence of varying maltodextrin concentrations. On *M. smegmatis* WT, Douge exhibits the plaque-forming efficiency of 10^{-7} to 10^{-8}, with no significant reduction across maltodextrin concentrations. While, on the Δ*lamH* mutant, plaque formation occurs at the similar efficiency (10^{-7} to 10^{-8}) but decreases in a concentration-dependent manner with increasing maltodextrin levels.

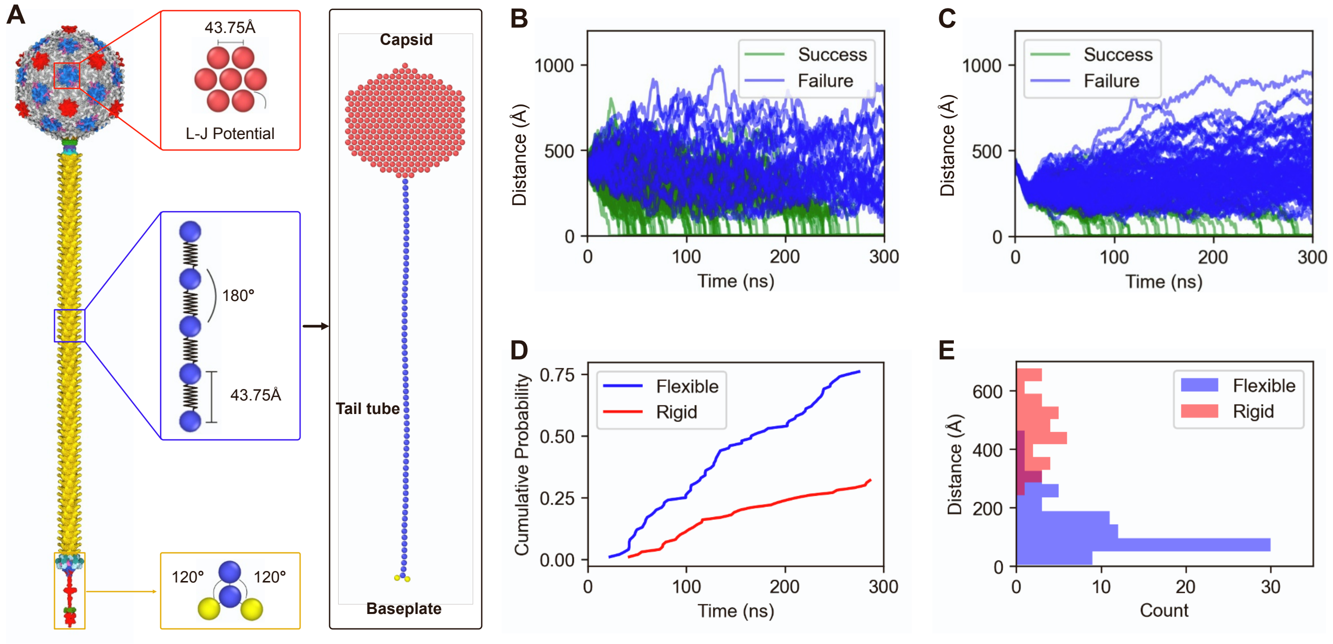

Figure S12. Coarse-grained Molecular Dynamics Simulation of Mycobacteriophage Douge.
(A) Schematic representation of the coarse-grained modeling pipeline for the capsid, tail tube, and baseplate, with the fully assembled coarse-grained model of Douge shown (left panel).
(B, C) Time evolution of the distance between the baseplate and the cell membrane over 100 simulations for the flexible tail (B) (see Video S2) and rigid tail (C) models (see Video S3), showing how each configuration approaches the membrane during the simulation.
(D) Distribution of baseplate binding times, showing that the flexible tail binds more efficiently and reaches the membrane faster than the rigid tail.
(E) Comparison of the capsid-to-membrane distance at the moment of baseplate binding, showing differences between flexible and rigid tail models

Table S1. Cryo-EM single particle refinements and validation statistics for different phage components.

Data collection	Dataset-1 (12,214 movies) | Dataset-02 (18,201 movies) | Dataset-03 (14,199 movies)									
Cryo-EM equipment	Titan Krios									
Voltage (kV)	300									
Cs (mm)	2.7									
Magnification (nominal)	81,000									
Gun lens	4									
Spot size	4									
Detector	K3									
Detector (Operation mode)	Super resolution									
Dose rate (e^{-}/Å^2 per second)	~ 24.2									
Pixel size (Å/pixel) (Super resolution)	0.5305									
Electron exposure (e^{-}/Å^2)	~ 48.40									
Exposure time (s)	2.0									
Frames (no.)	50									
Defocus range (μm)	-0.43 ~ -2.97									
Grid type	R2/1 (2 nm C-film)									
Reconstruction	**Genome–packed**						**Genome–free**			
	Capsid (I) (EMD-39973) (PDB:8ZDH)	Connector (C6) (EMD-39983) (PDB:8ZDJ)	Vertex (C5) (EMD-39984) (PDB:8ZDK)	Tail tube (C6) (EMD-60024) (PDB:8ZEA)	Baseplate (C3) (EMD-39996) (PDB:8ZDO)	Central fiber (C3) (EMD-60001) (PDB:8ZDP)	Capsid (I) (EMD-39978) (PDB:8ZDI)	Connector (C6) (EMD-39989) (PDB:8ZDL)	Vertex (C5) (EMD-39990) (PDB:8ZDM)	Tail tube (C6) (EMD-39995) (PDB:8ZDN)
**Software**	**CryoSPARC**									
Micrographs stacks (no.)	44,614	44,614	44,614	30,415	44,614	44,614	44,614	44,614	44,614	954
Final particle images (no.)	94,629	28,210	28,210	1,591,245	78,927	31,169	21,074	3,700	3,700	12,010
Symmetry imposed	I	C6	C5	C6	C3	C3	I	C6	C5	C6
Map final resolution (Å)†	3.20	3.18	3.44	2.18	2.97	4.26	3.58	3.77	4.26	3.07
Map sharpening B-factor (Å^2)	-133.3	-86.3	-67.4	-89.0	-91.0	-138.5	-145.2	-61.6	-58.3	-73.5
Atomic modeling										
**Software**	**Coot and Phenix**									
Number of protein residues	3,513	11166	11,570	8,970	11,289	2,289	3,417	11,154	11,280	8,970
Number of metal ions	0	0	0	0	0	0	0	0	0	0
Number of atoms	26,513	87468	87,290	67,680	86,910	17,019	25,757	87,390	85,025	67,680
Map CC (around atoms)	0.87	0.86	0.88	0.89	0.84	0.80	0.86	0.83	0.83	0.87
RMSD bond lengths (Å)	0.008	0.002	0.006	0.003	0.004	0.002	0.004	0.002	0.002	0.002
RMSD bond angles (°)	1.072	0.447	0.553	0.552	0.527	0.484	0.974	0.437	0.505	0.513
Clash score	6.52	7.06	8.00	7.45	8.28	13.58	6.45	8.32	8.87	6.85
Ramachandran favored (%)	97.19	95.66	96.32	99.47	97.42	95.88	97.44	96.36	94.97	98.63
Ramachandran allowed (%)	2.80	4.34	3.68	0.53	2.58	4.12	2.56	3.64	4.56	1.37
Ramachandran outliers (%)	0	0	0	0	0	0	0	0.00	0.47	0
Rotamer outliers (%)	0	0	0	0	0	0	0	0	0	0
C_β deviations	0	0	0	0	0	0	0	0	0	0
MolProbity score	1.51	1.69	1.68	1.41	1.56	1.93	1.47	1.69	1.82	1.38

†According to FSC=0.143

Table S2. DALI search results for Douge RBPs

Douge Protein-domains	PDB ID (chain)	Z-score	Rmsd (Å)	Alignment identity (%)	Properties
MCP-Ig-like	8gyr (B)	13.2	1.1	26	Leptospira immunoglobulin-like protein
	7r3t (A)	12.7	1.2	30	Beta-1,3-glucanase BGLH
	7z4f (G)	10.1	1.8	22	Putative structural protein
	8f2m (D)	10.0	2.4	10	Major capsid protein
	4uj6 (A)	9.8	2.0	26	Surface layer protein
TTP-Ig-like	8gyr (B)	13.3	1.3	27	Leptospira immunoglobulin-like protein
	7r3t (A)	12.3	1.5	38	Beta-1,3-glucanase BGLH
	4uj6 (A)	10.2	2.0	29	Surface layer protein
	7z4f (G)	10.1	1.8	22	Putative structural protein
	8opr (A)	9.9	2.5	17	S-layer protein ea1
CFP-NTD	2zew (B)	14.2	2.6	16	S-layer associated multidomain endoglucanase
	8iqe (A)	13.3	2.5	14	K2-vcl6 tsp
	8xmz (A)	12.8	2.3	13	T9ss c-terminal target domain-containing protein
	4d0q (A)	12.8	2.4	18	Hyaluronate lyase
	8hhv (A)	12.7	2.4	18	Endo-alpha-d-arabinanase
CFP–tip domain	7toh (A)	8.8	3.2	6	Sgnh hydrolase
	3iii (A)	8.7	3.5	10	COCE/NOND family hydrolase
	8yzo (A)	8.1	3.3	9	Lipase
	3gbw (A)	8.1	3.4	9	E3 ubiquitin-protein ligase mycbp2
	7zvo (A)	7.8	2.6	11	Beta-galactosidase
CFP-CTD	4azz (A)	17.0	2.5	11	Levanase
	5n8k (A)	16.9	2.8	8	Galactocerebrosidase
	1bjq (B)	15.0	2.9	14	Lectin
	8jp4 (A)	14.9	3.0	11	Protein ergic-53
	1oq1 (A)	14.9	2.6	12	Protein yesu
BppU-NTD	7eeb (E)	7.1	2.7	10	Enhanced green fluorescent protein, cation channel
	6sws (A)	6.6	2.8	8	Phosphoinositide 3-kinase adapter protein 1
	3tp4 (B)	6.6	4.0	4	Computational design of enzyme
	7dm0 (A)	6.5	2.4	17	Biofilm-associated surface protein
	8oe4 (D)	6.3	3.5	7	Interleukin-12 subunit beta
BppU-CBM	5n8k (A)	17.6	2.8	9	Galactocerebrosidase
	4azz (A)	16.5	2.5	9	Levanase
	8jp4 (A)	15.4	3.0	13	Protein ergic-53
	5zbt (A)	14.7	3.3	11	Lectin-like protein
	1bjq (B)	14.6	2.8	11	Lectin

Table S3. Detailed interactions at various interfaces among connector, tail tube, and baseplate proteins.

Portal – Adaptor (4:1)	
**Portal**	**Adaptor**
L264^{p2}	V39^{a1}<br>V40^{a1}
**D267^{p1}**	**R104^{a1}**
**S265^{p1}**	**K106^{a1}**
**E226^{p1}**	**G109^{a1}**
F295^{p1}	L111^{a1}
**M287^{p1}**	**M112^{a1}**
D263^{p2}	M112^{a1}
**I279^{p1}**	**T113^{a1}**
**I285^{p1}**	**V114^{a1}**
K277^{p2}	S115^{a1}
**D283^{p1}**	**T116^{a1}**
**L278^{p2}**	
**L280^{p2}**	**S117^{a1}**
**E54^{p3}**	
**D243^{p4}**	**R118^{a1}**
**H249^{p3}**	**E120^{a1}**
**I48^{p3}**	**E121^{a1}**
**Y46^{p4}**	**R123^{a1}**
**E42^{p3}**	**K128^{a1}**
**K31^{p4}**	**T129^{a1}**
**R40^{p3}**	**Y131^{a1}**
**Y120^{p4}**	**Y134^{a1}**
R29^{p3}	D136^{a1}
**R146^{p3}**	**D136^{a1}**

Adaptor – Stopper (3:1)	
**Adaptor**	**Stopper**
F82^{a1}	V30^{s1}
P81^{a4}	T37^{s1}
M79^{a1}	Y60^{s1}
F82^{a1}	W89^{s1}
Q78^{a2}	H93^{s1}
F82^{a3}	P94^{s1}
V84^{a3}	F95^{s1}
**R77^{a2}**	**D96^{s1}**
R77^{a2}	F98^{s1}
M79^{a2}	F100^{s1}

Stopper – Terminator (1:3)	
**Stopper**	**Terminator**
Y9^{s1}	I46^{t1}
**N15^{s1}**	**S33^{t2}**<br>**C35^{t2}**<br>**W36^{t2}**
K16^{s1}	N18^{t2}
**K16^{s1}**	**R82^{t1}**<br>**S81^{t1}**
**N18^{s1}**	**R82^{t1}**<br>**D83^{t1}**
K21^{s1}	D133^{t1}
**R46^{s1}**	**T127^{t3}**
K49^{s1}	W36^{t1}
**K49^{s1}**	**A37^{t1}**<br>**L125^{t3}**
R72^{s1}	D133^{t1}
**E80^{s1}**	**E45^{t1}**<br>I46^{t1}

TTP – TTP (Intra-ring–1:1)	
**TTP**	**TTP**
F4^{i1}	N20^{i6}<br>T22^{i6}<br>M144^{i6}
I7^{i1}	I46^{i6}<br>L24^{i6}
**K8^{i1}**	**L21^{i6}**<br>**S52^{i6}**
**Q11^{i1}**	**Q99^{i6}**
**L14^{i1}**	**N100^{i6}**
**I16^{i1}**	**I117^{i6}**
**E37^{i1}**	**S119^{i6}**
P39^{i1}<br>T40^{i1}	F121^{i6}
**K58^{i1}**	**D173^{i6}**
**L62^{i1}**	**L171^{i6}**
**L64^{i1}**	**Q196^{i6}**
**P79^{i1}**	**K88^{i6}**
**E80^{i1}**	**R187^{i6}**
**T84^{i1}**	**N134^{i6}**
**R89^{i1}**	**I108^{i6}**
W156^{i1}	I117^{i6}
**V195^{i1}**	**A128^{i6}**
**F199^{i1}**	**I124^{i6}**
**W204^{i1}**	**G122^{i6}**
**R205^{i1}**	**F121^{i6}**
**A211^{i1}**	**R102^{i6}**
**G212^{i1}**	**R102^{i6}**
F213^{i1}	I124^{i6}<br>R102^{i6}

TTP – TTP (Inter-ring–1:3)	
**TTP**	**TTP**
I73^{i1}	V176^{i6}
**E74^{i1}**	**K58^{i6}**<br>**Y98^{i6}**
A75^{i1}	H55^{i6}
**Y76^{i1}**	**P18^{i6}**<br>L21^{i6}<br>**F56^{i6}**<br>**K58^{i6}**<br>N172^{i5}<br>D173^{i5}
**E78^{i1}**	**H55^{i6}**<br>**K58^{i6}**
**R83^{i1}**	**D145^{i1}**
I85^{i1}	L19^{i1}

TTP – DTP (1:3)	
**TTP**	**DTP**
K71^{k1}	Y122^{d2}
E74^{k1}<br>**A75^{k1}**<br>**E78^{k1}**	**K124^{d1}**
Y76^{k1}	
**Y76^{k1}**	**D125^{d1}**<br>**L31^{d2}**<br>L35^{d2}<br>**Y39^{d2}**
P79^{k1}	I128^{d2}
**R83^{k1}**	**D131^{d2}**
V131^{k1}	I128^{d3}

DTP – BHP (1:2/2:1)	
**DTP**	**BHP**
P49^{d2}	H496^{h2}
**Y56^{d2}**	**K39^{h2}**
**M198^{d2}**	**K41^{h2}**
**Y199^{d2}**	**R62^{h2}**
**R228^{d2}**	**R88^{h2}**
Q230^{d2}	Y92^{h2}
G246^{d2}	Y59^{h2}
**D248^{d2}**	**R62^{h2}**
**K44^{d3}**	E136^{h2}<br>**H137^{h2}**
L46^{d3}<br>Y47^{d3}<br>V48^{d3}	V138^{h2}<br>R88^{h2}
**K59^{d3}**	**E376^{h3}**
W176^{d3}	W368^{h3}
**Y199^{d3}**	**R364^{h3}**
**H220^{d3}**	**N3^{h3}**
**S222^{d3}**	**R31^{h3}**
**Q230^{d3}**	W499^{h3}
**N236^{d3}**	**S349^{h3}**
**R243^{d3}**	**Q342^{h3}**
**K245^{d3}**	**F316^{h6}**
D248^{d3}	R364^{h3}
C274^{d3}	F20^{h3}
**K275^{d3}**	**E24^{h3}**
Q350^{d3}	W499^{h3}

BHP – CFP (2:1)	
**BHP**	**CFP**
**T239^{h2}**<br>**D258^{h2}**<br>**W238^{h2}**	**M1^{f2}**<br>**T2^{f2}**<br>**M3^{f2}**
Y323^{h2}<br>F327^{h2}<br>R479^{h2}<br>A177^{h2}	P4^{f2}<br>L10^{f2}
**L472^{h2}**<br>**A474^{h2}**	**D11^{f2}**
F170^{h2}	W19^{f2}
**G175^{h2}**<br>**A177^{h2}**<br>**D204^{h2}**<br>**N160^{h2}**	**N21^{f2}**<br>**S26^{f2}**<br>**L28^{f2}**
F170^{h2}	V36^{f2}
D427^{h3}	R37^{f2}
F432^{h3}	F40^{f2}
**Y412^{h3}**<br>**G417^{h3}**	**Q45^{f2}**
F418^{h3}	**W50^{f2}**
**I403^{h2}**	

Bold-Orange: H-bonds; Yellow: Electrostatic interactions; Gray: Hydrophobic / Cation–pcontacts

Table S4. Binding of individual RBPs to mycobacterial glycan array* (structure of glycans are on next page)

Glycans	His-GST-MCP-Ig-like domain	His-MBP-TTP-Ig-like domain	His-CFP-NTD	His-CFP-tip-CTD	His-BppU-NTD	His-GST-BppU-CBM
S1	0	0	272	0	0	0
S2	0	0	0	0	0	0
S3	0	0	0	0	0	0
S4	0	0	0	0	0	0
S5	0	0	0	0	0	0
S6	0	0	0	0	0	0
S7	780	0	315	1647	0	0
S8	2010	1	746	5191	388	0
S9	1074	0	722	2997	377	0
S10	2018	0	1425	2	0	0
S11	2295	251	3383	4378	842	0
S12	0	0	0	0	0	0
S13	2077	0	652	4725	145	0
S14	2850	86	572	5758	568	0
S15	2041	184	2067	5062	945	0
S16	2469	31	1178	3619	483	0
S17	331	15	1180	20	349	0
S18	5489	733	10605	12094	2233	0
S19	9641	1205	14679	31017	2853	1461
S20	4324	389	4403	13412	1638	0
S21	6809	1076	15014	19746	2622	1230
S22	7745	956	15995	15520	2271	1382
S23	1544	167	2594	2766	1063	0
S24	5404	809	7744	12820	1998	0
S25	5571	623	6609	18151	1587	0
S26	0	0	0	0	0	0
S27	0	0	176	0	40	0
S28	180	0	0	0	0	0
S29	0	0	0	0	0	0
S30	0	0	0	0	0	0
S31	0	0	0	0	0	0
S32	0	0	0	0	0	0
S33	0	0	0	0	0	0
S34	0	0	0	0	0	0
S35	0	0	0	0	0	0
S36	0	0	0	0	0	0
S37	0	0	91	0	0	0
S38	0	0	0	7325	0	0
S39	0	0	0	22426	0	0
S40	0	0	0	0	0	0
S41	0	0	0	0	0	0
S42	0	0	0	0	0	0
S43	0	0	0	0	0	0
S44	0	0	0	0	0	0
S45	0	0	0	0	0	0
S46	1642	19841**	1563	6240	220	0
S47	0	0	0	0	0	0
S48	1192	226	1418	3146	645	0
S49	595	148	1275	3512	103	0
S50	2250	319	2497	6718	1008	0
S51	0	0	0	0	0	0
S52	1289	33992**	2389	5474	403	0
S53	0	0	0	0	0	0
S54	0	0	0	0	0	0
S55	0	0	0	0	0	0
S56	0	0	0	0	0	0
S57	0	0	268	0	0	0
S58	2621	222	3155	8977	348	0
S59	0	0	0	0	0	0
S60	0	0	0	0	0	0

*Numbers are average MFI of three replicate measurements.
**Presumed binding to MBP-Fusion tag

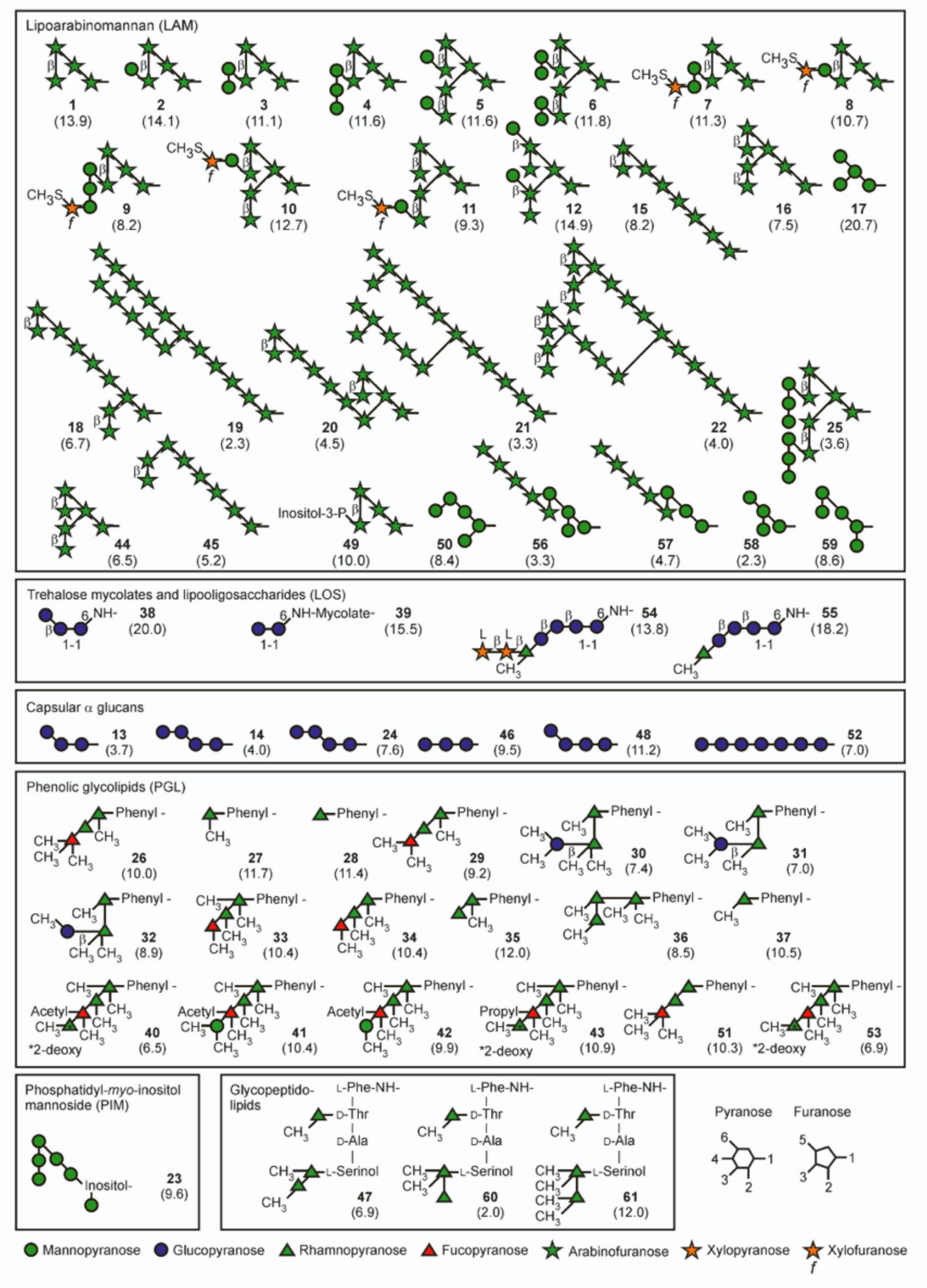

Lipoarabinomannan (LAM)
1 (13.9)
2 (14.1)
3 (11.1)
4 (11.6)
5 (11.6)
6 (11.8)
7 (11.3)
8 (10.7)
9 (8.2)
10 (12.7)
11 (9.3)
12 (14.9)
15 (8.2)
16 (7.5)
17 (20.7)
18 (6.7)
19 (2.3)
20 (4.5)
21 (3.3)
22 (4.0)
25 (3.6)
44 (6.5)
45 (5.2)
Inositol-3-P
49 (10.0)
50 (8.4)
56 (3.3)
57 (4.7)
58 (2.3)
59 (8.6)
Trehalose mycolates and lipooligosaccharides (LOS)
38 (20.0)
NH-Mycolate-
39 (15.5)
54 (13.8)
55 (18.2)
Capsular α glucans
13 (3.7)
14 (4.0)
24 (7.6)
46 (9.5)
48 (11.2)
52 (7.0)
Phenolic glycolipids (PGL)
26 (10.0)
27 (11.7)
28 (11.4)
29 (9.2)
30 (7.4)
31 (7.0)
32 (8.9)
33 (10.4)
34 (10.4)
35 (12.0)
36 (8.5)
37 (10.5)
40 (6.5)
41 (10.4)
42 (9.9)
43 (10.9)
51 (10.3)
53 (6.9)
*2-deoxy
Phosphatidyl-myo-inositol mannoside (PIM)
Inositol-
23 (9.6)
Glycopeptido-lipids
L-Phe-NH-
D-Thr
D-Ala
L-Serinol
47 (6.9)
60 (2.0)
61 (12.0)
Pyranose
Furanose
Mannopyranose
Glucopyranose
Rhamnopyranose
Fucopyranose
Arabinofuranose
Xylopyranose
Xylofuranose

Table S5. Force field parameters of the coarse–grained model.

m_h	233434.34amu	θ_{02}	90°
m_t	17734.375amu	θ_{03}	120°
m_{te}	17734.375amu	σ_{ht}	38.977Å
m_w	100000amu	σ_{hte}	38.977Å
m_p	233434.34amu	σ_{hw}	51.683Å
σ_h	38.977Å	σ_{hp}	38.977Å
σ_t	38.977 Å	σ_{tte}	38.977Å
σ_{te}	38.977 Å	σ_{tw}	51.683Å
σ_w	68.531 Å	σ_{tp}	38.977Å
σ_p	38.977 Å	σ_{tew}	51.683Å
ε_h	100kcal/mol	σ_{tep}	38.977Å
ε_t	0.001 kcal/mol	σ_{wp}	51.683Å
ε_{te}	0.001 kcal/mol	ε_{ht}	0.316 kcal/mol
ε_w	0.1 kcal/mol	ε_{hte}	0.316 kcal/mol
ε_p	100 kcal/mol	ε_{hw}	0.3 kcal/mol
k_t	3.762 kcal/mol /Å^2	ε_{hp}	1 kcal/mol
δ_0	43.75 Å	ε_{tte}	0.001 kcal/mol
T	1200K	ε_{tw}	0.01 kcal/mol
ρ	36.44%	ε_{tp}	0.0316 kcal/mol
$k_{\theta 1}$	1250 kcal/mol	ε_{tew}	0.01 kcal/mol
$k_{\theta 2}$	1250 kcal/mol	ε_{tep}	100 kcal/mol
$k_{\theta 3}$	1250 kcal/mol	ε_{wp}	0.3 kcal/mol
θ_{01}	180°		
